# Redefining the competitive paradigm: influence of co-occurring ions on PFAS adsorption from polluted water onto granular activated carbon and anion exchange resin

**DOI:** 10.1007/s11356-026-38084-1

**Published:** 2026-07-28

**Authors:** Mehak Fatima, Celine Kelso, Faisal I. Hai

**Affiliations:** 1 Strategic Water Infrastructure Laboratory, School of Engineering, University of Wollongong, Wollongong, NSW 2522 Australia; 2 Molecular Horizons, School of Science, University of Wollongong, Wollongong, NSW 2522 Australia

**Keywords:** Per- and polyfluoroalkyl substances (PFAS), Inorganic ions, Shorter-chain PFAS (PFPeA)

## Abstract

The effects of four inorganic ions: magnesium chloride (MgCl_2_), potassium nitrate (KNO_3_), sodium phosphate (Na_2_PO_4_), sodium bicarbonate (NaHCO_3_), and three organic ions: sodium dodecyl sulfate (SDS), ethylenediaminetetraacetic acid (EDTA), and humic acid (HA) on the adsorption behaviour of five per- and polyfluoroalkyl substances (PFAS) with varying chain lengths (C_5_–C_11_) were systematically investigated using granular activated carbon (GAC) and anion exchange resin (AER). Pseudo-second order (PSO) kinetic modelling showed adsorption capacities (qₑ) ranged from 1.11 to 6.89 mg/g and 4.67 to 8.62 mg/g for GAC and AER, respectively, across all experimental conditions and controls. AER consistently maintained high PFAS removal across diverse water matrices, whereas GAC performance was influenced by PFAS molecular structure and background ion composition. Competitive adsorption among PFAS followed a chain length–dependent trend on GAC; however, distinct selectivity patterns were observed for both adsorbents, highlighting material-specific adsorption mechanisms. Slight pH variations from background water are unlikely to destabilize PFAS adsorption, indicating stable performance of adsorption-based treatment under realistic conditions. Notably, this work reveals a previously unreported ion-mediated enhancement in the adsorption of short- and medium-chain PFAS (perfluoropentanoic acid, PFPeA; perfluorononanoic acid, PFNA) on GAC, attributed to cation-bridging and strengthened electrostatic interactions. In contrast, the longest-chain PFAS (perfluoroundecanoic acid, PFUnDA) exhibited a reversed response, with suppressed adsorption in the presence of ions. Moreover, desorption experiments revealed that co-occurring ions can enhance the retention of highly mobile, shorter-chain PFAS (PFPeA) on adsorbents, limiting PFAS remobilization, thereby improving the long-term reliability of adsorption-based water treatment systems. Overall, these findings provide new mechanistic insight into PFAS-adsorbent interactions under realistic water matrix conditions and offer practical guidance for adsorbent selection and treatment optimization especially for shorter-chains that are more problematic.

**Graphical abstract:**

**Figure:**
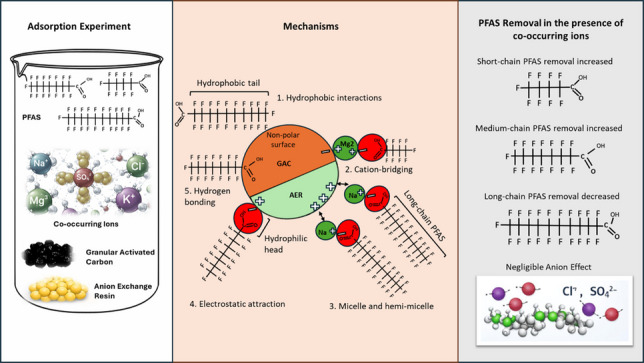

**Supplementary Information:**

The online version contains supplementary material available at 10.1007/s11356-026-38084-1.

## Introduction

Per- and polyfluoroalkyl substances (PFAS) are a large group of synthetic organofluoride compounds characterized by strong carbon-fluorine (C-F) bonds and amphiphilic molecular structures, which together confer exceptional environmental persistence and mobility in aquatic systems (Bentel et al. 2019; Buser and Morf 2009; Stahl et al. 2011). PFAS are fluorinated surfactants comprising of perfluoroalkyl acids (PFAAs), including perfluorinated carboxylic acids (PFCAs) and perfluoroalkane sulfonates (PFSAs) (Wang et al. 2017) and are therefore extensively used in firefighting foams, paints, non-stick cookware, waterproof apparel, and leather products (Bečanová et al. 2016; Favreau et al. 2017; 3M C 1999). As a result, PFAS are ubiquitously detected in surface waters, groundwater, wastewater effluents, and drinking water supplies worldwide (Du et al. 2014; Kwok et al. 2015). In real environmental and engineered water matrices, PFAS rarely occur as single contaminants; instead, they coexist with a complex mixture of organic matter and inorganic ions that can substantially influence their transport, fate, and removal behaviour.

At environmentally relevant pH values, most PFAS exist as anions (Fatima et al. 2025), making electrostatic interactions a dominant factor governing their behaviour in water treatment and natural systems. Inorganic ions such as chloride (Cl⁻), nitrate (NO₃⁻), sulfate (SO₄^2^⁻), bicarbonate (HCO₃⁻), and phosphate (PO₄^3^⁻) can compete directly with PFAS for adsorption sites on engineered adsorbents, particularly anion exchange resins (AER) and mineral surfaces (Du et al. 2014, 2015). Increased ionic strength can also compress the electrical double layer surrounding charged surfaces, reducing electrostatic repulsion and, in some cases, enhancing PFAS adsorption onto hydrophobic media such as activated carbon (Yu et al. 2009).

Divalent cations, including calcium (Ca^2^⁺) and magnesium (Mg^2^⁺), introduce additional complexity to PFAS behaviour. These ions can promote cation-bridging between negatively charged PFAS molecules and negatively charged adsorbent surfaces or organic matter, potentially enhancing PFAS retention despite increased ionic strength (Higgins and Luthy 2006). Such effects are highly system-specific and depend on PFAS chain length, functional group chemistry, and the surface properties of the adsorbent (Fatima et al. 2025). Consequently, the presence of divalent ions may lead to non-monotonic or non-linear adsorption trends that are poorly captured by single-solute models.

Natural organic matter (NOM) and other dissolved organic constituents represent another major class of co-occurring species influencing PFAS behaviour (Levchuk et al. 2018). Organic macromolecules such as humic and fulvic acids can compete with PFAS for adsorption sites, block pores within adsorbent materials, or modify surface chemistry through pre-adsorption and fouling effects (Liu et al. 2019; Sahara et al. 2024). In addition, hydrophobic interactions between PFAS fluorinated chains and organic matter may promote association or complexation, increasing apparent PFAS mobility while reducing effective adsorption onto engineered media (Hamza et al. 2025). These interactions are particularly relevant in wastewater-impacted waters, where elevated organic loads coexist with trace-level PFAS.

Matrix effects are especially pronounced for short-chain PFAS, which exhibit higher aqueous solubility and weaker hydrophobic interactions than their long-chain counterparts. In multicomponent systems, short-chain PFAS are frequently displaced by longer-chain PFAS or by organic co-solutes with stronger affinities for adsorption sites, leading to preferential retention of long-chain compounds and reduced removal of short-chain species (McCleaf et al. 2017). This behaviour poses a significant challenge for treatment systems, particularly as regulatory and industrial trends increasingly favour short-chain PFAS replacements (Zhang et al. 2023).

Despite extensive research on PFAS adsorption and removal, much of the existing mechanistic understanding is derived from single-solute batch studies conducted in ultrapure water. Such simplified systems fail to capture competitive adsorption, synergistic interactions, and site heterogeneity that arise under realistic water quality conditions (Cousins et al. 2020). Consequently, predictions based on single-compound isotherms often fail to predict adsorption capacity and treatment performance when applied to complex matrices, particularly at environmentally relevant concentrations (Fatima et al. 2026).

As regulatory limits for PFAS continue to decrease globally, there is a growing need to understand how co-occurring organic and inorganic constituents modify PFAS behaviour in natural and engineered water systems. Additionally, the regulatory attention is increasingly shifting toward short-chain PFAS; it is essential to better understand the competitive interactions occurring within PFAS groups themselves as well as the co-solutes. Hence, within this context, our suite spans short‑chain (PFPeA), medium‑chain (PFOA, PFNA, PFDA), and long‑chain (PFUnDA) PFAS, allowing us to systematically evaluate chain length effects on the adsorption and desorption behaviour. PFPeA represents the emerging short‑chains that are increasingly detected but less well characterized, while PFUnDA extends the list towards longer‑chain PFAS that exhibit stronger sorption and persistence, and are frequently reported in environmental monitoring studies (USEPA 2024). Together, these five PFAS provide direct relevance to current and evolving regulatory frameworks. They also cover both legacy and replacement chemistries. Therefore, the primary objective of this study was to develop a mechanistic understanding of the adsorption mechanisms of a range of PFAS chain length on granular activated carbon (GAC) and AER by (i) investigating the competitive adsorption behaviour between short-, medium-, and long-chain PFAS in multi-solute systems; (ii) examining the influence of inorganic and organic ions, as well as varying PFAS initial concentrations, on the absorption of PFAS; and (iii) assessing the influence of inorganic and organic ions on the desorption of PFAS.

## Materials and methods

### Chemicals and materials

Perfluoropentanoic acid (PFPeA 97% purity), perfluorooctanoic acid (PFOA 95% purity), perfluorononanoic acid (PFNA 97% purity), perfluorodecanoic acid (PFDA 98% purity), and perfluoroundecanoic acid (PFUnDA 95% purity) were purchased from Sigma-Aldrich (Melbourne, Victoria). Amberlite HPR4811 CL AER was also purchased from Sigma-Aldrich. GAC-GS1300 was received from Activated Carbon Technologies Pty Ltd. (Melbourne, Victoria). Methanol, MeOH (HPLC grade), acetone (HPLC grade, magnesium chloride (MgCl_2_), potassium nitrate (KNO_3_), sodium phosphate (Na_2_PO_4_), sodium bicarbonate (NaHCO_3_), sodium dodecyl sulfate (SDS), ethylenediaminetetraacetic acid (EDTA), and humic acid (HA) used in this study were all purchased from Sigma-Aldrich (Melbourne, Victoria). The physical and chemical properties of the five PFAS used in this study are provided in the Table 1.

**Table 1:**
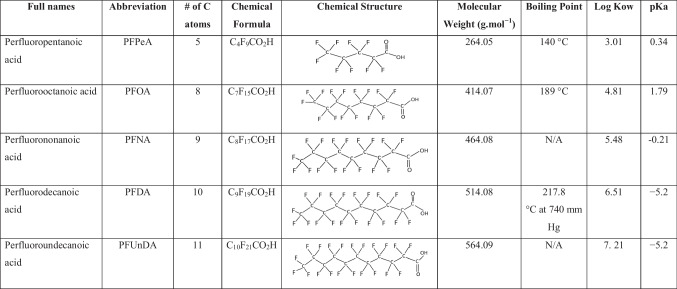
Chemical and physical properties of target compounds used in this study (N/A indicates data not available) (Fatima et al. 2026)

### Standard solutions and working solutions preparation

The selection of five PFAS (PFPeA, PFOA, PFNA, PFDA, and PFUnDA) was guided by both regulatory relevance and the need to capture a representative range of perfluoroalkyl chain lengths from PFPeA (C_5_) to PFUnDA (C_11_). PFOA and PFNA are explicitly addressed in recent regulatory frameworks, including the U.S. EPA National Primary Drinking Water Regulation, where they are individually regulated in hazard index–based mixtures due to their occurrence and health concerns (Grunfeld et al. 2024; USEPA 2024). Stock solutions of individual PFAS were prepared following our previous study (Fatima et al. 2026). Briefly, the stock and standard solutions were prepared in MeOH, based on the preliminary solvent evaluation study included in the supplementary material (Fig. S1). MeOH was selected because it ensured complete solubility of all target PFAS and provided stable ionization under negative mode ESI. Three hundred microliters of calibration standards was prepared using MeOH-based stocks which were subsequently diluted with Milli‑Q water. Dilution with Milli-Q ensured that the calibration standards closely resembled the experimental matrix.

### Adsorbent preparation

This study employed two types of adsorbents: GAC and AER as noted in section. The adsorbents were prepared as described in our previous study (Fatima et al. 2026). Details of the characteristics of both the adsorbent are provided in the supporting information (Tables S1 and S2).

### Batch adsorption experiments

Batch adsorption and kinetic experiments were conducted following our previous study on PFAS isotherm (Fatima et al. 2026). Briefly, a series of adsorption isotherm experiments was conducted to evaluate the influence of co-occurring inorganic and organic ions on PFAS removal. Experimental conditions were standardized across all trials, with PFAS mixture concentrations ranging from 5 to 25 mg/L, adsorbent dosage of 50 mg, a working volume of 25 mL, agitation at 150 rpm, temperature maintained at 28 ± 1 °C, and an initial pH of 3.7 ± 0.2 over a 24 h equilibration period. Four inorganic ions, MgCl_2_, KNO_3_, Na_2_PO_4_, and NaHCO_3_, were each tested at 1 mM, while three organic constituents, SDS, EDTA, and HA, were evaluated at 1 mg/L. Co-occurring ions were selected to represent commonly co‑existing compounds typically present in natural and engineered water matrices. Multivalent cations (e.g. Mg^2^⁺) and monovalent ions (e.g. Na⁺ and K⁺) are common in groundwater and surface waters, while phosphate and bicarbonate are common anions (Wood et al. 2022). The selected concentration of 1 mM for inorganic salts lies within the lower end of environmentally relevant ranges reported for natural waters, where major ions commonly occur between ~ 0.1 and 5 mM depending on geology and catchment characteristics (Umeh et al. 2023). The organic constituents were selected to represent typical organic matrix components: SDS as an anionic surfactant, EDTA as a strong chelating agent (Mousavi and Khodadoost 2019), and humic acid as a representation of natural organic matter. Their concentrations (1 mg/L) fall within the range observed for dissolved organic species in surface waters and treated effluents. In total, seven isotherm experiments were performed, with each interfering species assessed individually across four initial PFAS concentrations (5, 10, 15, and 25 mg/L). The 5 mg/L condition was performed in duplicate to assess experimental reproducibility, while the remaining concentrations were run once each. The full dataset for 5 mg/L duplicates and 10–25 mg/L kinetic profiles are provided in Supplementary Table S3 and Fig S2-S4, respectively. PFAS concentrations (5–25 mg/L) exceed typical environmental levels to ensure measurable adsorption kinetics and generate observable adsorption trends across different concentrations. The initial pH of 3.7 ± 0.2 resulted from the addition of PFAS to Milli‑Q water. Because PFAS are acids, their addition naturally lowered the pH of the solution. No pH adjustment was performed, to maintain a matrix free of external buffers that could interfere with adsorption, particularly in the presence of the seven co-occurring ions evaluated in this study. Hence, to carefully assess the ion‑specific interactions, the solution pH was left unmodified. Periodic sampling was performed at six intervals (0, 1, 3, 6, 9, 12, and 24 h) to capture adsorption kinetics and equilibrium behaviour under each interference condition.

### Desorption experiment

After estimating the PFAS loading capacity of GAC and AER through adsorption, the desorption of PFAS from the adsorbent was a concern. To address this concern, subsequent desorption experiments were conducted to determine the maximum amount of PFAS that could be released into the solvent over 24 h. The experiment aimed to evaluate whether desorption behaviour differed among PFAS species and in the presence of different co-occurring ions. The PFAS-loaded adsorbent was separated from the aqueous phase and transferred into 25 mL of MeOH. The suspensions were agitated under same experimental conditions to the adsorption experiments to promote solvent extraction of PFAS from the adsorbent. The mixtures were then analyzed for PFAS using LC–MS. The amount of PFAS desorbed was expressed as qe,_des_ (mg/g), and desorption efficiency (%) was calculated relative to the amount initially adsorbed. MeOH was selected as the extraction solvent based on previously done recovery experiments (Fig. S1).

### Analytical method

PFAS preparation and LC–MS/MS analysis followed the validated procedures from our previous study (Fatima et al. 2026), with details on instrumental parameters, calibration standards, quality controls, and limits of detection (LoDs) and quantification (LoQs) provided in the supporting information (Tables S4-S6). Briefly, Samples were collected in 300-µL polypropylene (PP) vials pre‑loaded with 25 µL of 25% acetonitrile to ensure complete recovery of PFAS during analysis, followed by vortexing for at least 30 s to fully desorb any PFAS adsorbed to analytical vial surfaces. To evaluate the adsorption kinetics, pseudo-second-order (PSO) models (Table S7) that assume chemical reaction were applied to fit the kinetic data using Eq. (2). Additionally, chi‑square (χ^2^) values were calculated from Eq. (2) to identify the model fitting of the data. χ^2^ represented the difference between the experimental results and the model predictions (Table S7), similar to the role of the coefficient of determination (R^2^). A χ^2^ value close to zero indicated that the calculated values were in good agreement with the experimental data.

1 $$PSO:\;\frac1{q_t}=(\frac1{K_2Q_e^2})(\frac1t)+(\frac1{q_e})$$

2 $${x}^{2}= \sum \frac{{\left({qe}_{exp}- {qe}_{cal}\right)}^{2}}{{qe}_{cal}}$$

where q_e,exp_ (mg/g) is the experimentally measured adsorption capacity at equilibrium. q_e_,_cal_ (mg/g) is the adsorption capacity predicted by the PSO kinetic model. Σ represents the summation over all data points used in the model fit.

### QA/QC analysis

The LC–MS/MS method provided robust sensitivity and linearity for all target PFAS, with LoD values between 0.01 to 0.1 mg/L and LoQ values between 0.2 to 0.4 mg/L, and calibration coefficients (*R*^*2*^) ranging from 0.97 to 0.99 (Table S6). Comparison of chromatograms for standards prepared in water and in MeOH at the same concentrations showed that MeOH‑based standards (subsequently diluted with Milli‑Q) consistently produced higher peak intensities and more defined signals (Fig S5a-d). This is consistent with the greater solubility of PFAS in MeOH, which reduces adsorption losses to vial surfaces and improves transfer into the mobile phase, thereby enhancing recovery. MeOH blanks prepared at the same dilution ratio as the working standards, together with Milli‑Q blanks run before and between samples, did not show significant PFAS peaks above LoD, apart from minor carryover signals for PFPeA and PFOA in the water blank immediately following standard injections (Fig S5c). These low signals (< LoQ) did not affect quantification in the experimental samples. Hence, MeOH was selected as the solvent for stock and working solutions, followed by dilution in Milli‑Q to match the aqueous analytical conditions, providing an accurate balance between PFAS solubility, recovery, and reliable calibration performance.

## Results and discussion

### Comparative adsorption kinetics of PFAS onto GAC and AER in the presence of co-occurring ions

**Fig. 1:**
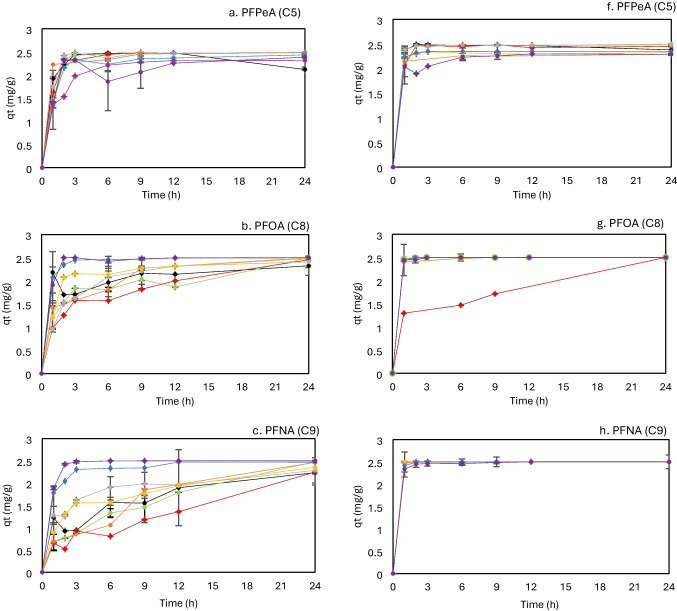

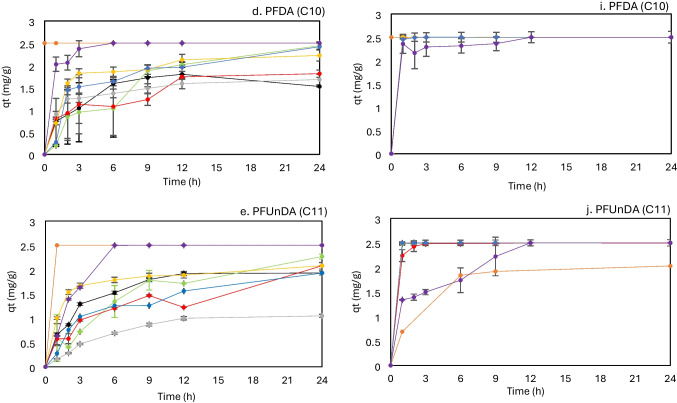
Batch adsorption kinetics of five PFAS in the presence of seven co-occurring ions (colour coded as follows: PFAS-HA (green), PFAS-EDTA (red), PFAS-SDS (grey), PFAS-MgCl_2_ (orange), PFAS-KNO_3_ (yellow), PFAS-Na_3_PO_4_ (purple), and PFAS-NaHCO₃ (blue) along the controls (black) expressed as adsorbed concentration (q_t_, mg/g) at a given time (t, h) on GAC (**a**–**e**) and AER (**f**–**j**) with C_o_ 5 mg/L. Constant parameters include pH 3.7 ± 0.2, temperature 28 ± 1 °C, and agitation speed 150 RPM. Error bars represent standard deviation from duplicate experiments

The adsorption kinetics of five PFAS compounds (PFPeA, PFOA, PFNA, PFDA, and PFUnDA) onto GAC and AER were evaluated at four different initial concentrations (5, 10, 15, and 25 mg/L) in the presence of seven co‑occurring ions (HA, EDTA, SDS, KNO₃, MgCl₂, Na₃PO₄, NaHCO₃). Kinetic profiles are shown in Fig. 1a–j, while the complete dataset from the duplicates at 5 mg/L and kinetic profiles across all concentrations (10–25 mg/L) are provided in the supplementary information Table S3 and Fig. S2-S4, respectively.

Across all PFAS, adsorption on GAC (Fig. 1a–e) and AER (Fig. 1f–j) in most systems reached equilibrium within 6–9 h and 6 h, respectively. However, the extent and rate of uptake varied depending on both PFAS chain length and the solution matrix. The PFAS‑only controls (CNT) exhibited the slowest and most gradual adsorption profiles, providing a consistent baseline for the evaluation of ions effect. For the short‑ and mid‑chain PFAS (PFPeA, PFOA, PFNA), majority of ions enhanced adsorption kinetics relative to the control on both adsorbents, producing steeper early‑time slopes and earlier stabilization.

On GAC, the kinetic profile for PFPeA followed NaHCO₃ ≈ MgCl₂ ≈ SDS ≈ HA > KNO₃ > EDTA > Na₃PO₄ > control, while for PFOA and PFNA, the ion‑containing systems clustered closely and were uniformly faster than the control (Fig. 1a–c). A similar pattern was observed on AER, where PFPeA again showed the strongest ion‑dependent acceleration, with the general trend NaHCO₃ ≈ HA ≈ SDS > MgCl₂ > KNO₃ > control > Na₃PO₄ > EDTA, and PFOA and PFNA again displayed uniform kinetics across all ions (Fig. 1f–h). Similarly, PFDA exhibited minimal ion influence on both adsorbents (Fig. 1d and i): all ion‑containing systems rose rapidly and nearly overlapped except SDS, indicating that PFDA adsorption did not experience noticeable competition or kinetic suppression from any ion. These results are consistent with previous findings showing that long-chain PFAS are less susceptible to competition from co-occurring ions (Campos Pereira et al. 2018; Zhang et al. 2023). However, SDS significantly decreased adsorption of PFUnDA (longest‑chain PFAS) on GAC, relative to the control. Overall PFDA and PFUnDA followed a similar trend. These combined results demonstrate a clear chain length–dependent shift in ion influence: all ions enhance adsorption for PFPeA–PFNA, exert negligible competition for PFDA, and reduce adsorption for PFUnDA, with almost consistent trends across both GAC and AER.

Additionally, the adsorption kinetics were evaluated using PSO models that revealed the adsorption capacities (q_e_). q_e_ varied between 1.11–6.89 mg/g for GAC and 4.67–8.62 mg/g for AER across all experimental conditions and controls. The PSO model provided a good fit to the experimental data, as evidenced by high correlation coefficients (*R*^*2*^) and closer agreement (*χ*^*2*^) between experimental and predicted q_e_ values (Table S7). This suggests that the adsorption process is predominantly governed by surface interactions and adsorption capacity rather than simple diffusion-controlled processes (Fatima et al. 2026).

### Effect of PFAS initial concentration

**Fig. 2:**
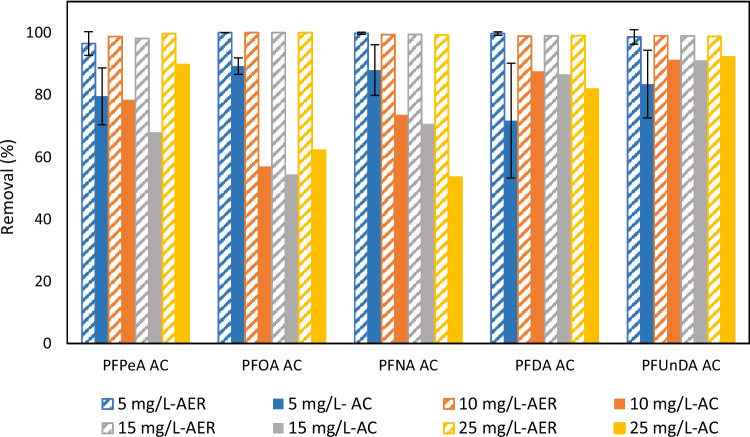
Removal (%) of PFAS on GAC (expressed as AC, solid bars) and AER (broken/hatched bars) as a function of initial PFAS concentration. Error bars represent standard deviation from triplicate experiments

Given the wide variability of PFAS concentrations in natural waters, it is important to understand how such fluctuations influence adsorption performance. Therefore, the effect of varying PFAS concentrations (5, 10, 15, and 25 mg/L) on the removal performance was tested. Removal performance for both adsorbents was strongly influenced by the initial PFAS concentration, and the trends differed markedly between GAC and AER (Fig. 2). For GAC, increasing PFAS concentration led to a decline in removal efficiency across all PFAS except PFPeA and PFOA where the removal increased as the concentration increased from 15 to 25 mg/L; this could be an experimental error. At lower concentrations (5–10 mg/L), GAC achieved moderate removal, but performance dropped substantially at 15–25 mg/L (Fig. 2). These results are consistent with reports that PFAS diffusion into GAC micropores becomes increasingly hindered as concentration increases. This results in reduced mass transfer driving force at higher solute loadings and reduced accessibility of internal adsorption sites (Fatima et al. 2026; Lei et al. 2023).

In contrast, AER exhibited minimal sensitivity to initial PFAS concentration, maintaining high removal (> 90%) across the entire concentration range (Fig. 2). This stability indicates rapid mass transfer of the compounds onto the AER, as it has strong affinity and the abundance of readily accessible adsorption sites (Fatima et al. 2026; Maimaiti et al. 2018; Umeh et al. 2023).

Overall, the contrasting behaviours of GAC and AER highlight the importance of adsorbent-PFAS interaction mechanisms. GAC relies primarily on hydrophobic interactions and pore‑filling (Deng et al. 2012; Du et al. 2014; Higgins and Luthy 2006), both of which are concentration‑dependent and susceptible to competitive effects. AER, however, removes PFAS through ion‑exchange processes that remain effective across a wide concentration range. As a result, AER demonstrates better stability to varying concentration, making it a more reliable option for treatment scenarios where PFAS levels fluctuate or influent concentrations are high. Details of removal percentages are provided in supplementary information (Table S8).

### Effect of co-occurring ions on PFAS adsorption

**Fig. 3:**
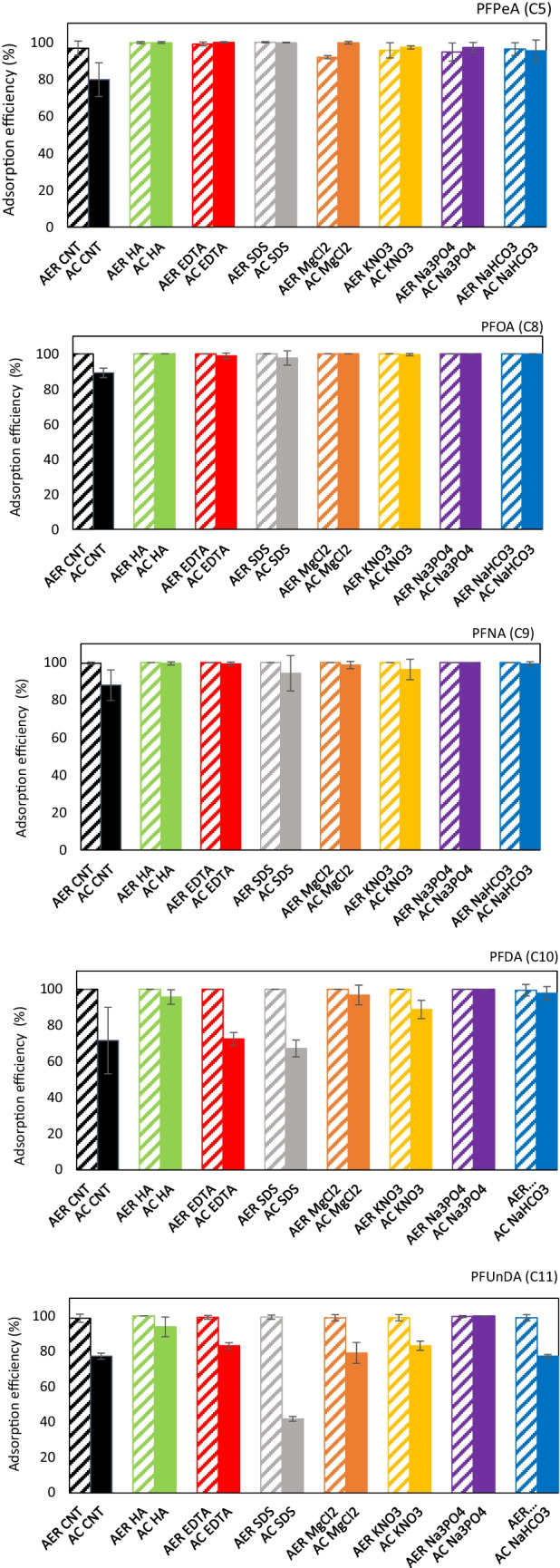
Adsorption efficiency percentage (%) of PFAS on AER (broken/hatched bars) and GAC (expressed as AC, solid bars) in the presence of co-occurring organic (EDTA, SDDA, HA) and inorganic ions (MgCl₂, KNO₃, Na₃PO₄, and NaHCO₃). Error bars represent standard deviation from triplicate experiments

As co-occurring ions are ubiquitous in natural waters, their effect on PFAS adsorption onto GAC and AER was evaluated which revealed that across all tested PFAS and solution matrices, AER consistently exhibited equal or higher removal compared to GAC (Fig. 3). The adsorption performance of AER remained relatively stable in the presence of co-occurring organic and inorganic ions, with only minor variations observed among different water chemistries. The stability of PFAS uptake on AER under complex matrices is attributed to strong electrostatic binding, surface charge neutralization, electrical double layer (EDL) compression, competitive adsorption, salting-out effects, and ion-paring interactions between anionic PFAS and quaternary ammonium functional groups (–NR₄⁺) on the resin surface (Boudreau et al. 2003; Du et al. 2014; Du et al. 2015; Gong et al. 2016; Wang and Shih 2011; You et al. 2010). This uniform behaviour suggests that ion exchange–driven adsorption onto AER was not significantly disrupted by competing ions, in agreement with prior studies reporting high selectivity of quaternary ammonium functional groups towards PFAS (Carter and Farrell 2010; Du et al. 2014).

In contrast, GAC showed greater variability in adsorption capacity depending on PFAS type and water matrix, consistent with earlier observations that carbon-based adsorbents are more sensitive to solution chemistry effects (Appleman et al. 2014; Yu et al. 2009). GAC exhibited chain length–dependent responses to co-occurring ions. Adsorption of short- to medium-chain PFAS (PFPeA, PFOA, PFNA) improved or remained stable (Fig. 3) as also observed in a similar study by Du et al. (2015). This trend shows that while molecular level interactions between PFAS and GAC surfaces are important, solution chemistry also substantially influences PFAS adsorption. In contrast, longer-chain PFAS (PFDA and PFUnDA) showed reduced adsorption capacity in the presence of certain background ions particularly in the presence of SDS (Fig. 3); similar inhibitory effects have been attributed to competitive adsorption, pore blockage, and reduced accessibility of adsorption sites for larger PFAS molecules (Appleman et al. 2014; Yu et al. 2009). Details on the adsorption mechanisms for all ions are discussed in the sections.

Overall, through this study, the effect of a diverse set of seven ions (four inorganic and three organic) was investigated, an insight that goes beyond the limited ion combinations typically reported in the literature and offers a more realistic representation of natural and engineered water systems. Details on the calculated adsorption efficiency (%) for all ions are provided in Table S9.

### Effect of co-occurring ions on PFAS desorption

**Fig. 4:**
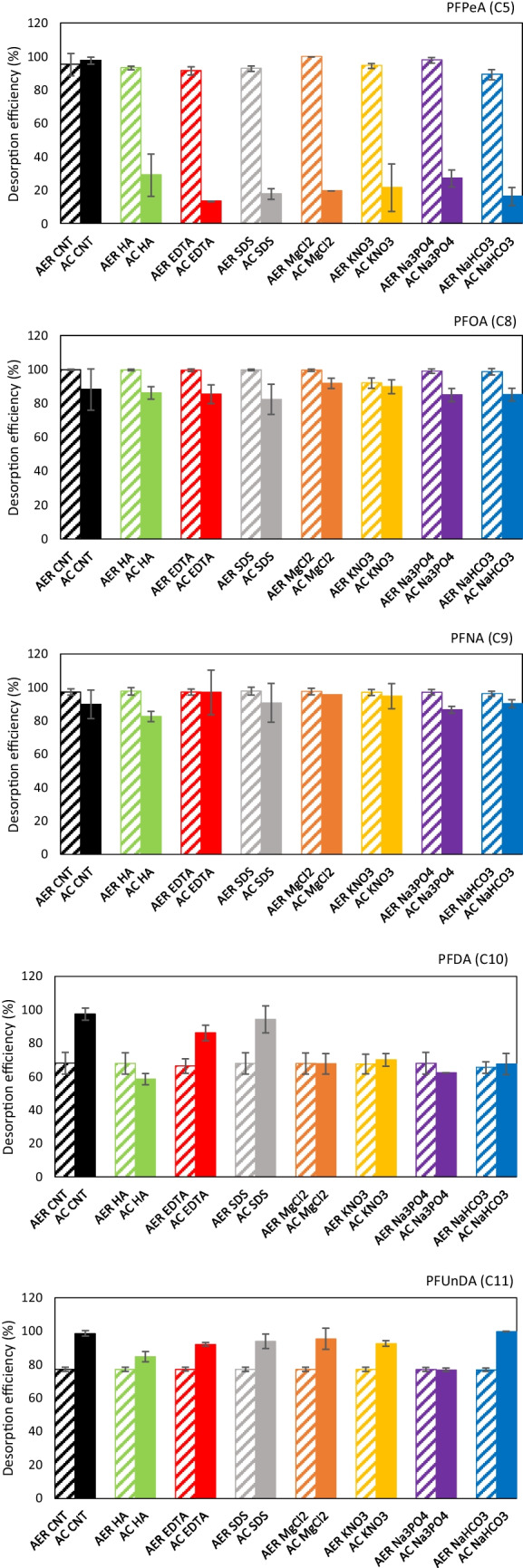
Desorption efficiency (%) of five PFAS in the presence of seven co-occurring ions (colour coded as follows: PFAS-HA (green), PFAS-EDTA (red), PFAS-SDS (grey), PFAS-MgCl_2_ (orange), PFAS-KNO_3_ (yellow), PFAS-Na_3_PO_4_ (purple), and PFAS-NaHCO₃ (blue) along the controls (black) on GAC (expressed as AC, solid bars) and AER (broken/hatched bars). Constant parameters include pH 3.7 ± 0.2, temperature 28 ± 1 °C, and agitation speed 150 RPM and 100% Methanol as the extraction solvent. Error bars represent standard deviation from duplicate experiments

Following adsorption, desorption of PFAS from the adsorbent is a concern (Nakazawa et al. 2024). Hence, the reversibility of adsorption was evaluated using desorption efficiency (%) (Table S10). Desorption (%) represents the fraction of adsorbed PFAS released during the desorption step. Results presented in Fig. 4 show that the presence of co-occurring ions influenced PFAS desorption from GAC in a compound-specific manner, with the most pronounced effect observed for the short-chain, PFPeA. In comparison to the control experiment (desorption ~ 100%), the presence of co-solutes reduced PFPeA desorption following the order: HA > KNO₃ > Na₃PO₄ > SDS > NaHCO₃ > EDTA. This reduction suggests that the presence of dissolved organic matter and co-occurring ions may promote stronger retention of shorter-chain PFAS on the adsorbent surface, potentially through electrostatic interactions, or enhanced hydrophobic partitioning. A similar phenomenon has previously been reported for the perfluorobutanoic acid (PFBA, C_4_) (Nakazawa et al. 2024). The desorption ratio of PFBA, despite it being the most hydrophilic and least strongly adsorbed compound, remained unexpectedly low in that study (Nakazawa et al. 2024). PFBA exhibited much earlier breakthrough compared to NOM. This indicates that the migration of the mass transfer zone (MTZ) through the GAC bed occurred more rapidly for PFBA than for most NOM components. This suggests that NOM adsorption predominantly took place after PFBA had already been adsorbed. The subsequent adsorption of NOM likely contributed to PFBA desorption to some extent. NOM may also have significantly led to pore blockage at the entrance of micropores where PFBA was initially retained due to its small molecular size. Once the entrance pathways were obstructed, PFBA was trapped within the pore structure, limiting further displacement by competing solutes and thereby reducing its overall desorption (Nakazawa et al. 2024). However, reports describing such behaviour for short-chain PFAS remain extremely limited.

In this study, the desorption behaviour of PFOA and PFNA remained largely unaffected by the presence of co-occurring ions, with desorption efficiencies comparable to the control conditions. For the longer chains (PFDA and PFUnDA), desorption again decreased in the presence of co-solutes, with the largest reductions observed in systems containing HA and Na₃PO₄. This behaviour likely reflects the stronger hydrophobic interactions and greater adsorption affinity of long-chain PFAS. This can be further stabilized by the presence of background ions and organic matter.

On the other hand, the desorption behaviour on AER showed minimal sensitivity to co-occurring ions. Desorption (%) for PFPeA, PFOA, and PFNA remained approximately 100% across all conditions. However, PFDA and PFUnDA exhibited lower desorption (~ 70–80%) across all experiments, including the controls, indicating stronger retention of longer chains on the resin. This resulted in a clear chain length–dependent trend on AER, where desorption decreased with increasing PFAS chain length (Fig. 4).

Overall, this study represents one of the first systematic evaluations of the effect of co-occurring ions on PFAS desorption behaviour from commonly used adsorbents. While numerous studies have investigated the influence of water matrix constituents on PFAS adsorption, their role in controlling the subsequent desorption and potential remobilization of PFAS has received limited attention. The findings are particularly relevant for short-chain PFAS, which are generally considered more difficult to retain during treatment due to their higher mobility and lower adsorption affinity compared to long-chain analogues. The observed reduction in PFPeA desorption in the presence of several co-occurring ions suggests that background water chemistry may actually contribute to stabilizing the retention of these highly mobile PFAS on adsorbents. This insight has important implications for long-term water treatment performance. Hence, these findings indicate that naturally occurring organic matter and inorganic ions may help limit PFAS remobilization and improve the reliability of adsorption-based treatment systems.

### Effect of co-occurring ions on solution pH

**Fig. 5:**
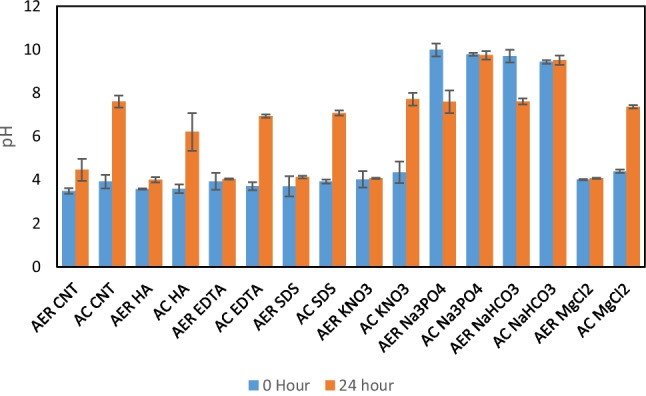
Initial (0 h) and final (24 h) solution pH measured during adsorption experiments of five PFAS in the presence of seven co-occurring ions: HA, EDTA, SDS MgCl_2_, KNO_3_, Na_3_PO_4_, and NaHCO₃ along the controls on GAC (expressed as AC) and AER. Error bars represent the standard deviation from duplicate measurements

The influence of co-occurring ions on solution pH is important to consider as pH fluctuations in natural water may cause variable adsorption patterns. Therefore, pH before and after the adsorption experiments was evaluated and the results are presented in Fig. 5. Across most experimental conditions, an increase in pH was observed after 24 h of contact with the adsorbents. This trend was particularly pronounced in the GAC systems, where the final pH increased substantially in several matrices, including the control. The increase in pH can be attributed to the release of basic surface functional groups or the consumption of protons during interactions between PFAS molecules and the adsorbent surface. Activated carbon is known to have oxygen-containing functional groups that can influence solution pH through surface proton exchange processes (Bernal et al. 2018). In contrast, the pH changes observed in the AER systems were generally smaller, indicating a comparatively lower influence of the resin matrix on bulk solution chemistry. Moreover, certain co-occurring ions also contributed to distinct pH shifts. For example, Na₃PO₄ and NaHCO₃ resulted in alkaline conditions due to their buffering capacity.

The observed pH variations did not appear to promote PFAS desorption from either adsorbent. Despite the increase in pH under several experimental conditions, particularly in the presence of Na₃PO₄ and NaHCO₃, adsorption efficiencies did not increase and in some cases were further reduced. This behaviour can be explained by the fact that PFAS remain predominantly in their anionic form across the pH range observed in this study. Consequently, the adsorption and retention of PFAS were significantly influenced by adsorbent-PFAS interactions and the presence of co-occurring ions. These findings suggest that moderate pH fluctuations arising from background water chemistry are unlikely to destabilize PFAS adsorption, supporting the stability of adsorption-based treatment processes under realistic water matrix conditions. Details on the initial (0 h) and final (24 h) solution pH measured during adsorption experiments for all ions are provided in Table S11.

### PFAS adsorption mechanisms

#### Ion-mediated uptake of short- and mid-chain PFAS

**Fig. 6:**
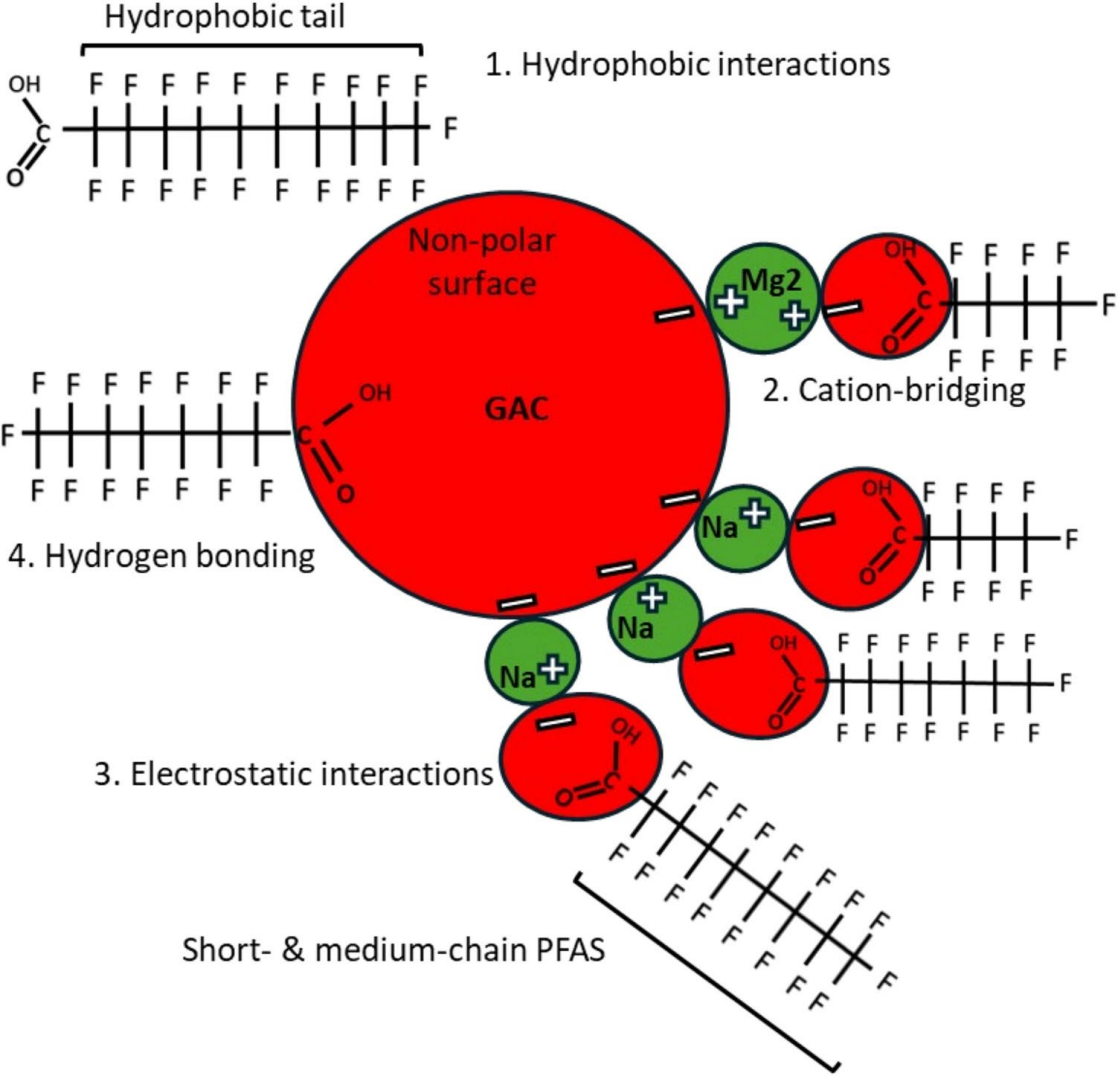
Different adsorption mechanisms of short- and medium-chain PFAS onto GAC

As distinct adsorption trends were observed for short- and mid-chain PFAS, further investigation into the governing mechanisms was undertaken. Across both GAC and AER, the ion‑dependent enhancement trends for short- and mid-chain PFAS adsorption can be explained by a common set of structural and interfacial mechanisms. The comparative analysis of co-occurring salts suggests that both cationic and anionic components may influence PFAS adsorption, although their individual contributions could not be independently examined within the current experimental design. For instance, in case of PFPeA removal on GAC, the experimental observations and comparison of KNO₃ and Na₃PO₄ based on anions reveal that despite the difference in anion valency, with NO₃⁻ being monovalent and PO₄^3^⁻ trivalent, both KNO₃ and Na₃PO₄ showed similar removal (97%). This suggests that anion-specific effects are relatively limited under the studied conditions. On the contrary, comparisons between cations (Na^+^ and K^+^) from the same compounds indicate that cations with similar charge may produce comparable effects. Accordingly, the higher removal observed in the presence of MgCl₂ (99%) points toward a potential role of cation valency (Mg^2^⁺). Divalent ions such as Mg^2^⁺ may enhance electrostatic interactions or bridging effects. Similar in the case of Na₃PO₄ and NaHCO_3_, Na^+^ increases the ionic strength and HCO₃⁻ from the later provides a mild buffering that reduces repulsion between anionic PFAS and negatively charged GAC surface (Adak et al. 2005; Dontsova and Bigham 2005; Lu et al. 2016). Therefore, MgCl_2_ and KNO_3_ provide moderate enhancement through their cationic contribution to ionic strength (Xiao et al. 2011). Additionally, divalent cations (Mg^2^⁺) have been observed to enhance PFAS adsorption not only through electrostatic screening but also through their strong water structuring (kosmotropic) behaviour (Welchert et al. 2024). Mg^2^⁺ possess a high charge density (Zangi et al. 2007) and carry large, tightly bound hydration shells, which strengthen the surrounding hydrogen‑bonding network (Jia et al. 2016; Liu et al. 2015; Mahmoudvand et al. 2019). This kosmotropic behaviour reduces the ability of water to solvate hydrophobic moieties (PFAS) and can promote their adsorption from bulk solution toward the solid-liquid interface (Liu et al. 2013). In this way, MgCl can indirectly increase PFAS affinity for both GAC and AER surfaces by creating a more structured interfacial water environment that favours adsorption. Hence, cation-bridging process creates positively charged surface sites that can attract anionic PFAS through electrostatic interactions (Fig. 6), thereby increasing adsorption capacity (Higgins and Luthy 2006; Liu et al. 2018; Lu et al. 2016).

Overall, with anion-specific effects appearing comparatively less significant under these conditions, the comparable performance across salts with varying cations points toward a relatively greater influence of cation. For example, enhanced removal in the presence of MgCl₂ indicates the influence of cation characteristics, particularly valency on adsorption behaviour. Therefore, the following discussion focuses on the role of cations while acknowledging that coupled ion effects cannot be entirely excluded.

Hence, unlike most previous studies that predominantly report inhibitory effects of co-occurring ions on PFAS adsorption due to competitive interactions, this study demonstrates a distinct chain length–dependent, ion-mediated enhancement in the uptake of short- and medium-chain PFAS.

#### Chain length–dependent reversed ion effects on the adsorption of long-chain PFAS

**Fig. 7:**
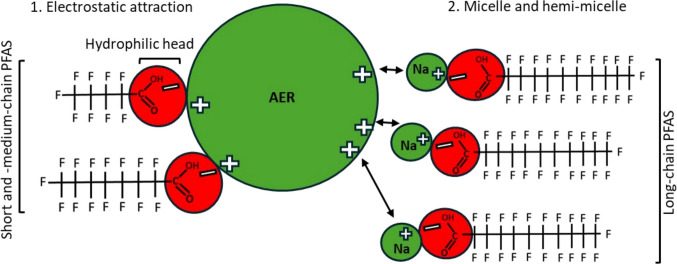
Different adsorption mechanisms of long-chain PFAS onto AER

Contrary to the trends observed for short- and mid-chain PFAS, long-chain PFAS displayed a reversed ion effect on adsorption, necessitating further investigation into the underlying chain length–dependent mechanisms. Generally, anionic PFAS can adsorb onto GAC and AER through hydrophobic interactions, van der Waals forces and electrostatic attractions (Figs. 6 and 7) (Chen et al. 2012, 2011; Fatima et al. 2025; Meyer et al. 2006; Yu et al. 2009). However, the extent to which solution chemistry influences this process depends strongly on chain length. Long‑chain PFAS such as PFUnDA rely predominantly on hydrophobic interactions to adsorb onto activated carbon (Deng et al. 2012). Changes in ionic composition can substantially alter interfacial water structure and double‑layer behaviour at the carbon surface. Therefore, the presence of ions produced a more pronounced effect on PFUnDA adsorption on GAC than on AER, where adsorption is governed mainly by strong electrostatic attraction to quaternary ammonium groups (Fatima et al. 2026; Lenka et al. 2024) and is therefore less sensitive to ion‑induced interfacial restructuring (Fig. 3).

Long-chain PFAS such as PFUnDA are known to form hemi-micelles and micelles in aqueous solutions due to hydrophobic interactions among their perfluorinated (C–F) chains (Du et al. 2014; Wang and Shih 2011; Yu et al. 2009). In this context, counterion hydration (i.e. the extent to which water molecules surround an ion) may influence the interaction of Na⁺, K⁺, and Mg^2^⁺ with PFUnDA aggregates, potentially promoting self-aggregation in solution (Fig. 3), a behaviour well established for surfactant-like compounds such as long-chain PFAS (Domínguez et al. 1997; Loganathan and Wilson 2022). Monovalent ions such as K⁺ appear to enhance this effect; owing to their lower hydration compared to divalent ions (Anacker and Ghose 1963), they may associate more closely with PFAS headgroups to contribute to PFAS self-aggregation as compared to the hydrated ones (Mg^2^⁺). Hence, the resulting PFUnDA aggregates could limit the diffusion of PFUnDA into the microporous structure of GAC, reducing access to internal adsorption sites and ultimately leading to decreased adsorption efficiency (Fig. 3).

Unlike GAC, AER adsorption is dominated by strong electrostatic ion exchange interactions (Fig. 7) that override ion-induced aggregation effects, resulting in stable PFUnDA removal even in the presence of ions. However, PFUnDA adsorption was slightly inhibited by HA (Fig. 3). HA is a highly polyanionic, surface‑active macromolecule (Li et al. 2025) with strong affinity for quaternary ammonium groups, enabling it to occupy a substantial fraction of the exchange sites and effectively block PFUnDA binding. This behaviour is consistent with previous studies showing that HA is one of the common inhibitors of PFAS removal by different adsorbents including AER, GAC, and soil due to competitive adsorption (Jeon et al. 2011; Li et al. 2025; Yang et al. 2016).

Overall, in contrast to the enhanced uptake observed for short- and medium-chain PFAS, this study further reveals a reversed ion effect for the longest-chain PFAS, characterized by suppressed adsorption in the presence of monovalent and divalent cations. Such chain length–dependent reversal behaviour has not been previously reported.

#### Limited influence of anions on PFAS adsorption

As discussed in the previous “Ion-mediated uptake of short- and mid-chain PFAS” section, in our system, comparatively minor role of anion (HCO₃⁻, Cl⁻, NO₃⁻, PO₄^3^⁻) and adsorption inhibition was observed. Since the PFAS are always anionic and the adsorbents span both positive (AER) and neutral/negative (GAC) surface charges, any anion‑driven effects would be expected to influence the two adsorbents in opposite ways. Instead, the consistent enhancement patterns across both indicate that the dominant interactions arise from the cations acting on the PFAS molecules, primarily through electrostatic screening and reduced repulsion rather than from anion-adsorbent surface interactions. PO₄^3^⁻, in particular, is known to bind strongly to cationic or metal‑associated sites and can disrupt favourable PFAS surface interactions (Wu et al. 2020).

While there is an extensive focus on cation-induced competitive effects in current literature (Higgins and Luthy 2006; Lei et al. 2023; Wang and Shih 2011), the role of background anions has received limited attention. This work provides one of the first comparative evaluations of cation versus anion effects, indicating the minimal influence of background anions on PFAS adsorption.

### Interplay of PFAS in a PFAS-only multicomponent adsorption system

While evaluation of the effect of co-occurring ions on PFAS adsorption is important, understanding the interplay and competitive interactions among PFAS themselves is equally critical for assessing treatment performance. Therefore, the current study also examined the extent of competition among the five PFAS by comparing their multi-solute PFAS‑only controls. The controls revealed a clear competitive hierarchy, with PFPeA, PFOA, and PFNA exhibiting the slowest adsorption and therefore the greatest susceptibility to enhancement when ions were introduced (black solid bars, Fig. 3), consistent with a study that reported the significant cationic effects on shorter-chain PFAS (Campos Pereira et al. 2018). Overall, GAC demonstrated higher adsorption capacities for longer-chain PFAS compared to shorter-chain analogues, in agreement with previously reported chain length–dependent behaviour of PFAS (Aly et al. 2018; Appleman et al. 2013; Fatima et al. 2026; Kim et al. 2024; Xiao et al. 2017). In contrast, the adsorption of PFAS on AER is independent of chain length (black broken/hatched bars, Fig. 3). The consistently high removal across all PFAS is attributable to the strong-base AER employed in this study, which contains trimethylammonium functional groups (–N⁺(CH₃)₃) that allows AER to remove PFAS primarily through electrostatic interactions between positively charged resin sites and negatively charged PFAS headgroups (Fatima et al. 2026; Lenka et al. 2024).

Although long-chain-dependent adsorption behaviour of PFAS is well documented (Appleman et al. 2014; Gellrich et al. 2012; Zaggia et al. 2016), this work advances current understanding by demonstrating the contrasting responses observed for GAC and AER under identical multi-solute conditions providing a mechanistic insight into hydrophobic versus electrostatic-driven adsorption and having direct implications for adsorbent selection in treatment design.

## Conclusion

This study reveals the PFAS adsorption behaviour in multicomponent systems, which is strongly governed by adsorbent type, PFAS chain length, and background ion composition. While AER exhibited consistently stable removal performance across diverse water matrices, GAC showed sensitivity to molecular structure and co-occurring ions. Notably, cation-mediated enhancement in the adsorption of short- and medium-chain PFAS was observed on GAC, attributed to cation-bridging and strengthened electrostatic interactions. Whereas the longest-chain PFAS exhibited a reversed response characterized by competition from co-solutes. In contrast, background anions were found to exert minimal influence on PFAS uptake. Desorption experiments further revealed that PFAS retention is not entirely reversible and is strongly influenced by chain length. Interestingly, the results indicate that short-chain PFAS such as PFPeA can be effectively retained in the presence of co-occurring species through strengthened adsorption interactions, suggesting that such matrix effects may be utilized to address the persistent challenge of poor removal of short-chain PFAS. Overall, these findings provide new mechanistic insight into ion-regulated PFAS adsorption and emphasize the importance of considering water matrix composition in treatment design.

## Supplementary Information

Below is the link to the electronic supplementary material.

ESM 1 (DOCX 27.6 MB)

## Data Availability

All data supporting the findings of this study are available within the paper and its Supplementary Information. Should any raw data files be needed in another format, they are available from the corresponding author upon reasonable request.
