# Supplementary material for: Redefining the competitive paradigm: influence of co-occurring ions on PFAS adsorption from polluted water onto granular activated carbon and anion exchange resin
Source: Environ Sci Pollut Res Int. 2026 Jul 28;33(24):12555–70. doi: 10.1007/s11356-026-38084-1 (PMC13469196; doi:10.1007/s11356-026-38084-1)
Supplement: Supplementary file 1 [file 11356_2026_38084_MOESM1_ESM.docx]

**Redefining the competitive paradigm: Influence of co-occurring ions on PFAS adsorption onto granular activated carbon and anion exchange resin**

Mehak Fatima^[1]^, Celine Kelso ^b^, Faisal I. Hai ^a^*

**Selection of solvent for PFAS extraction from the adsorbents**

The chromatograms in Fig. S1 illustrate the relative recovery of the target PFAS compounds under different solvent conditions. Among the tested solvents, 100% methanol (MeOH) exhibited the highest peak intensities for all PFAS, demonstrating the most effective extraction performance.

**(a)**

**(b)**

**(c)**

**(d)**

**Fig S1.** Total ion chromatograms (TICs) and extracted ion chromatograms (XICs) based comparison of PFAS recovery using different extraction solvents based on LC–MS chromatographic peak intensities. (a) 100% water, (b) 20% MeOH, (c) 50% MeOH, and (d) 100% MeOH.

**Table S1.** Characteristics of the GAC used in this study.

| Parameter | GS1300 |
| --- | --- |
| Apparent density (g/CC) | 0.32 - 0.36 |
| Surface area (BET m^2^/g min) | 1300 |
| Ash content (% max) | 15 |
| Iodine number (mg of I_2_/g min) | 1100 |
| Hardness Index (% min) | 85 |
| Moisture as packed (% max) | 3 |
| Particle size | 0.7 - 0.9 mm (8x30 mesh) |

**Table S2.** Characteristics of AER used in this study.

| Parameter | Amberlite HPR4811 Cl |
| --- | --- |
| Matrix | Gel |
| Functional group | Trimethylammonium |
| Particle density (g/mL) | 1.08 |
| Diameter (mm) | 0.55 ± 0.05 |
| Ion exchange capacity | ≥ 1.3 eq/L (Cl-form) |

**Table S3.** Experimentally measure adsorption capacity (q_e_, mg/g) of PFAS on GAC and AER in the presence of co-occurring organic (EDTA, SDDA, HA) and inorganic ions (MgCl₂, KNO₃, Na₃PO₄, and NaHCO₃). Constant parameters include C_o_ 5 mg/L, pH 3.7 ± 0.2, Temperature 28 ± 1 °C and agitation speed 150 RPM. The standard deviation is calculated from duplicate experiments.

| **GAC** | | | | | | | | | | | | | | | | |
| --- | --- | --- | --- | --- | --- | --- | --- | --- | --- | --- | --- | --- | --- | --- | --- | --- |
| Time | PFPeA Control | | PFPeA HA | | PFPeA EDTA | | PFPeA SDS | | PFPeA MgCl | | PFPeA KNO_3_ | | PFPeA Na_3_PO_4_ | | PFPeA NaHCO_3_ | |
|  | q_e_ | SD | q_e_ | SD | q_e_ | SD | q_e_ | SD | q_e_ | SD | q_e_ | SD | q_e_ | SD | q_e_ | SD |
| 0 | 0.000 | 0.000 | 0.000 | 0.000 | 0.000 | 0.000 | 0.000 | 0.000 | 0.000 | n/a | 0.000 | 0.000 | 0.000 | 0.000 | 0.000 | 0.000 |
| 1 | 1.925 | 0.000 | 1.464 | 0.635 | 1.636 | 0.000 | 1.753 | 0.206 | 2.229 | n/a | 1.417 | 0.012 | 1.402 | 0.110 | 1.353 | 0.055 |
| 2 | 2.229 | 0.000 | 2.358 | 0.000 | 2.321 | 0.000 | 2.421 | 0.000 | n/a | n/a | 2.162 | 0.000 | 2.328 | 0.000 | 1.533 | 0.000 |
| 3 | 2.445 | 0.000 | 2.426 | 0.000 | 2.315 | 0.000 | 2.484 | 0.000 | n/a | n/a | 2.336 | 0.000 | 2.329 | 0.000 | 1.980 | 0.000 |
| 6 | 2.469 | 0.002 | 2.453 | 0.023 | 2.447 | 0.037 | 2.370 | 0.063 | 2.334 | n/a | 2.232 | 0.136 | 1.861 | 0.629 | 2.212 | 0.138 |
| 9 | 2.481 | 0.009 | 2.487 | 0.000 | 2.482 | 0.007 | 2.486 | 0.003 | 2.456 | n/a | 2.352 | 0.008 | 2.063 | 0.349 | 2.278 | 0.006 |
| 12 | 2.479 | 0.000 | 2.465 | 0.000 | 2.471 | 0.000 | 2.471 | n/a | n/a | n/a | 2.368 | 0.000 | 2.258 | 0.000 | 2.313 | 0.000 |
| 24 | 2.121 | 0.009 | 2.484 | 0.009 | 2.488 | 0.005 | 2.489 | 0.005 | 2.487 | n/a | 2.437 | 0.013 | 2.386 | 0.014 | 2.316 | 0.140 |
| Time | PFOA Control | | PFOA HA | | PFOA EDTA | | PFOA SDS | | PFOA MgCl | | PFOA KNO_3_ | | PFOA Na_3_PO_4_ | | PFOA NaHCO_3_ | |
|  | q_e_ | SD | q_e_ | SD | q_e_ | SD | q_e_ | SD | q_e_ | SD | q_e_ | SD | q_e_ | SD | q_e_ | SD |
| 0 | 0.000 | 0.000 | 0.000 | 0.000 | 0.000 | 0.000 | 0.000 | 0.000 | 0.000 | n/a | 0.000 | 0.000 | 0.000 | 0.000 | 0.000 | 0.000 |
| 1 | 2.185 | 0.446 | 1.206 | 0.254 | 0.979 | 0.409 | 0.962 | 0.000 | 1.424 | n/a | 1.234 | 0.338 | 1.914 | 0.389 | 2.073 | 0.040 |
| 2 | 1.700 | 0.000 | 1.533 | 0.000 | 1.259 | 0.000 | 1.519 | 0.000 | n/a | n/a | 2.081 | 0.000 | 2.500 | 0.000 | 2.348 | 0.000 |
| 3 | 1.707 | 0.000 | 1.838 | 0.000 | 1.578 | 0.000 | 1.626 | 0.000 | n/a | n/a | 2.155 | 0.000 | 2.500 | 0.000 | 2.450 | 0.000 |
| 6 | 1.965 | 0.307 | 1.815 | 0.045 | 1.573 | 0.104 | 2.088 | 0.300 | 1.808 | n/a | 2.140 | 0.073 | 2.424 | 0.108 | 2.464 | 0.046 |
| 9 | 2.164 | 0.160 | 2.032 | 0.199 | 1.820 | 0.020 | 2.209 | 0.280 | 2.272 | n/a | 2.276 | 0.011 | 2.479 | 0.029 | 2.469 | 0.037 |
| 12 | 2.144 | 0.000 | 1.867 | 0.000 | 2.001 | 0.000 | 2.320 | 0.000 | n/a | n/a | 2.312 | 0.000 | 2.495 | 0.000 | 2.497 | 0.000 |
| 24 | 2.326 | 0.203 | 2.500 | 0.000 | 2.457 | 0.044 | 2.411 | 0.125 | 2.500 | n/a | 2.485 | 0.021 | 2.500 | 0.000 | 2.499 | 0.001 |
| Time | PFNA Control | | PFNA HA | | PFNA EDTA | | PFNA SDS | | PFNA MgCl | | PFNA KNO_3_ | | PFNA Na_3_PO_4_ | | PFNA NaHCO_3_ | |
|  | q_e_ | SD | q_e_ | SD | q_e_ | SD | q_e_ | SD | q_e_ | SD | q_e_ | SD | q_e_ | SD | q_e_ | SD |
| 0 | 0.000 | 0.000 | 0.000 | 0.000 | 0.000 | 0.000 | 0.000 | 0.000 | 0.000 | 0.000 | 0.000 | 0.000 | 0.000 | 0.000 | 0.000 | 0.000 |
| 1 | 1.220 | 0.716 | 0.667 | 0.181 | 0.655 | 0.374 | 1.275 | 0.203 | 0.696 | 0.032 | 0.887 | 0.226 | 1.887 | 0.034 | 1.787 | 0.031 |
| 2 | 0.926 | 0.000 | 0.764 | 0.000 | 0.521 | 0.000 | 1.262 | n/a | n/a | 0.000 | 1.303 | 0.000 | 2.423 | 0.019 | 2.043 | 0.000 |
| 3 | 0.939 | 0.000 | 0.877 | 0.000 | 0.924 | 0.000 | 1.621 | n/a | n/a | 0.000 | 1.556 | 0.000 | 2.481 | 0.026 | 2.307 | 0.000 |
| 6 | 1.564 | 0.341 | 1.331 | 0.094 | 0.808 | 0.073 | 1.912 | 0.232 | 1.058 | 0.044 | 1.564 | 0.061 | 2.498 | 0.003 | 2.333 | 0.128 |
| 9 | 1.552 | 0.440 | 1.458 | 0.268 | 1.178 | 0.045 | 1.981 | 0.260 | 1.849 | 0.080 | 1.764 | 0.106 | 2.500 | 0.000 | 2.342 | 0.044 |
| 12 | 1.897 | 0.852 | 1.790 | 0.000 | 1.358 | 0.000 | 1.961 | n/a | n/a | 0.000 | 1.940 | 0.000 | 2.500 | 0.000 | 2.473 | 0.038 |
| 24 | 2.235 | 0.275 | 2.482 | 0.025 | 2.249 | 0.255 | 2.285 | 0.284 | 2.466 | 0.049 | 2.360 | 0.155 | 2.500 | 0.000 | 2.471 | 0.034 |
| Time | PFDA Control | | PFDA HA | | PFDA EDTA | | PFDA SDS | | PFDA MgCl | | PFDA KNO_3_ | | PFDA Na_3_PO_4_ | | PFDA NaHCO_3_ | |
|  | q_e_ | SD | q_e_ | SD | q_e_ | SD | q_e_ | SD | q_e_ | SD | q_e_ | SD | q_e_ | SD | q_e_ | SD |
| 0 | 0.000 | 0.000 | 0.000 | 0.000 | 0.000 | 0.000 | 0.000 | 0.000 | 2.500 | 0.000 | 0.000 | 0.000 | 0.000 | 0.000 | 0.000 | 0.000 |
| 1 | 0.745 | 0.510 | 0.202 | 0.019 | 0.806 | 0.037 | 0.903 | 0.065 | 2.500 | 0.000 | 0.702 | 0.039 | 2.024 | 0.161 | 0.275 | 0.010 |
| 2 | 0.893 | 0.535 | 0.844 | 0.613 | 0.927 | 0.043 | 1.245 | 0.092 | n/a | n/a | 1.598 | 0.091 | 2.060 | 0.164 | 1.447 | 0.075 |
| 3 | 1.039 | 0.342 | 0.948 | 0.665 | 1.126 | 0.055 | 1.261 | 0.090 | n/a | n/a | 1.825 | 0.099 | 2.373 | 0.180 | 1.518 | 0.080 |
| 6 | 1.591 | 0.045 | 1.032 | 0.645 | 1.075 | 0.052 | 1.370 | 0.096 | 2.500 | 0.000 | 1.856 | 0.103 | 2.500 | 0.000 | 1.636 | 0.086 |
| 9 | 1.723 | 0.202 | 1.877 | 0.130 | 1.234 | 0.060 | 1.483 | 0.107 | 2.500 | 0.000 | 1.907 | 0.108 | 2.500 | 0.000 | 1.937 | 0.103 |
| 12 | 1.800 | 0.069 | 2.017 | 0.058 | 1.743 | 0.085 | 1.592 | 0.113 | n/a | n/a | 2.125 | 0.120 | 2.500 | 0.000 | 1.959 | 0.104 |
| 24 | 1.526 | 0.014 | 2.442 | 0.083 | 1.813 | 0.089 | 1.682 | 0.116 | 2.500 | 0.000 | 2.222 | 0.126 | 2.500 | 0.000 | 2.419 | 0.115 |
| Time | PFUnDA Control | | PFUnDA HA | | PFUnDA EDTA | | PFUnDA SDS | | PFUnDA MgCl | | PFUnDA KNO_3_ | | PFUnDA Na_3_PO_4_ | | PFUnDA NaHCO_3_ | |
|  | q_e_ | SD | q_e_ | SD | q_e_ | SD | q_e_ | SD | q_e_ | SD | q_e_ | SD | q_e_ | SD | q_e_ | SD |
| 0 | 0.000 | 0.000 | 0.000 | 0.000 | 0.000 | 0.000 | 0.000 | 0.000 | 0.000 | 0.000 | 0.000 | 0.000 | 0.000 | 0.000 | 0.000 | 0.000 |
| 1 | 0.664 | 0.227 | 0.591 | 0.479 | 0.576 | 0.038 | 0.163 | 0.006 | 2.500 | 0.000 | 1.003 | 0.028 | 0.635 | 0.008 | 0.274 | 0.007 |
| 2 | 0.866 | 0.018 | 0.403 | 0.000 | 0.578 | 0.062 | 0.283 | 0.012 | n/a | n/a | 1.537 | 0.047 | 1.389 | 0.017 | 0.759 | 0.008 |
| 3 | 1.290 | 0.028 | 0.722 | 0.000 | 0.961 | 0.031 | 0.468 | 0.015 | n/a | n/a | 1.668 | 0.055 | 1.636 | 0.021 | 1.033 | 0.015 |
| 6 | 1.522 | 0.034 | 1.343 | 0.327 | 1.204 | 0.273 | 0.692 | 0.021 | 2.500 | 0.000 | 1.788 | 0.056 | 2.499 | 0.002 | 1.258 | 0.002 |
| 9 | 1.795 | 0.040 | 1.781 | 0.202 | 1.467 | 0.041 | 0.869 | 0.030 | 2.500 | 0.000 | 1.868 | 0.058 | 2.500 | 0.001 | 1.259 | 0.026 |
| 12 | 1.920 | 0.042 | 1.715 | 0.000 | 1.223 | 0.448 | 0.999 | 0.033 | n/a | n/a | 1.887 | 0.060 | 2.500 | 0.000 | 1.559 | 0.021 |
| 24 | 1.930 | 0.044 | 2.268 | 0.052 | 2.078 | 0.044 | 1.048 | 0.033 | 2.500 | 0.000 | 2.078 | 0.066 | 2.500 | 0.000 | 1.928 | 0.025 |
| **AER** | | | | | | | | | | | | | | | | |
| Time | PFPeA Control | | PFPeA HA | | PFPeA EDTA | | PFPeA SDS | | PFPeA MgCl | | PFPeA KNO_3_ | | PFPeA Na_3_PO_4_ | | PFPeA NaHCO_3_ | |
|  | q_e_ | SD | q_e_ | SD | q_e_ | SD | q_e_ | SD | q_e_ | SD | q_e_ | SD | q_e_ | SD | q_e_ | SD |
| 0 | 0.000 | 0.000 | 0.000 | 0.000 | 0.000 | 0.000 | 0.000 | 0.000 | 0.000 | n/a | 0.000 | 0.000 | 0.000 | 0.000 | 0.000 | 0.000 |
| 1 | 2.373 | 0.049 | 2.376 | 0.078 | 2.073 | 0.384 | 2.380 | 0.018 | 2.148 | n/a | 2.212 | 0.027 | 2.037 | 0.197 | 2.236 | 0.067 |
| 2 | 2.488 | 0.017 | 2.472 | 0.000 | 2.465 | 0.000 | 2.464 | 0.000 | n/a | n/a | 2.304 | 0.000 | 1.897 | 0.000 | 2.320 | 0.000 |
| 3 | 2.483 | 0.024 | 2.472 | 0.000 | 2.467 | 0.000 | 2.449 | 0.000 | n/a | n/a | 2.340 | 0.000 | 2.048 | 0.000 | 2.352 | 0.000 |
| 6 | 2.468 | 0.001 | 2.477 | 0.002 | 2.447 | 0.030 | 2.481 | 0.005 | 2.263 | n/a | 2.323 | 0.056 | 2.234 | 0.075 | 2.347 | 0.006 |
| 9 | 2.472 | 0.003 | 2.482 | 0.007 | 2.484 | 0.004 | 2.474 | 0.004 | 2.254 | n/a | 2.298 | 0.028 | 2.259 | 0.073 | 2.349 | 0.001 |
| 12 | 2.466 |  | 2.467 | 0.000 | 2.424 | 0.000 | 2.476 | 0.000 | n/a | n/a | 2.295 | 0.000 | 2.299 | 0.000 | 2.352 | 0.008 |
| 24 | 2.381 | 0.111 | 2.500 | 0.019 | 2.454 | 0.005 | 2.478 | 0.007 | 2.291 | n/a | 2.325 | 0.031 | 2.289 | 0.018 | 2.349 | 0.000 |
| Time | PFOA Control | | PFOA HA | | PFOA EDTA | | PFOA SDS | | PFOA MgCl | | PFOA KNO_3_ | | PFOA Na_3_PO_4_ | | PFOA NaHCO_3_ | |
|  | q_e_ | SD | q_e_ | SD | q_e_ | SD | q_e_ | SD | q_e_ | SD | Ave qe | SD | q_e_ | SD | q_e_ | SD |
| 0 | 0.000 | 0.000 | 0.000 | 0.000 | 0.000 | 0.000 | 0.000 | 0.000 | 0.000 | n/a | 0.000 | 0.000 | 0.000 | 0.000 | 0.000 | 0.000 |
| 1 | 2.466 | 0.011 | 2.468 | 0.006 | 1.296 | 0.000 | 2.457 | 0.023 | 2.399 | n/a | 2.446 | 0.032 | 2.446 | 0.028 | 2.429 | 0.045 |
| 2 | 2.497 | 0.004 | 2.488 | 0.000 | 0.000 | 0.000 | 2.493 | 0.009 | n/a | n/a | 2.461 |  | 2.450 | 0.071 | 2.499 | 0.001 |
| 3 | 2.500 | 0.000 | 2.500 | 0.000 | 0.000 | 0.000 | 2.500 | 0.000 | n/a | n/a | 2.500 |  | 2.496 | 0.006 | 2.500 | 0.000 |
| 6 | 2.500 | 0.000 | 2.497 | 0.005 | 1.470 | 0.000 | 2.500 | 0.000 | 2.479 | n/a | 2.499 | 0.001 | 2.498 | 0.003 | 2.500 | 0.000 |
| 9 | 2.500 | 0.000 | 2.495 | 0.007 | 1.714 | 0.000 | 2.500 | 0.000 | 2.500 | n/a | 2.500 |  | 2.495 | 0.007 | 2.500 | 0.000 |
| 12 | 2.500 | 0.000 | 2.500 | 0.000 | 2.500 | 0.000 | 2.500 | 0.000 | n/a | n/a | 2.500 | 0.000 | 2.500 | 0.000 | 2.500 | 0.000 |
| 24 | 2.500 | 0.000 | 2.500 | 0.000 | 2.500 | 0.000 | 2.500 | 0.000 | 2.500 | n/a | 2.500 | 0.000 | 2.500 | 0.000 | 2.500 | 0.000 |
| Time | PFNA Control | | PFNA HA | | PFNA EDTA | | PFNA SDS | | PFNA MgCl | | PFNA KNO_3_ | | PFNA Na_3_PO_4_ | | PFNA NaHCO_3_ | |
|  | q_e_ | SD | q_e_ | SD | q_e_ | SD | q_e_ | SD | q_e_ | SD | q_e_ | SD | q_e_ | SD | q_e_ | SD |
| 0 | 0.000 | 0.000 | 0.000 | 0.000 | 0.000 | 0.000 | 0.000 | 0.000 | 0.000 | 0.000 | 0.000 | 0.000 | 0.000 | 0.000 | 0.000 | 0.000 |
| 1 | 2.488 | 0.008 | 2.500 | 0.000 | 2.480 | 0.000 | 2.498 | 0.002 | 2.485 | 0.000 | 2.498 | 0.003 | 2.337 | 0.173 | 2.418 | 0.010 |
| 2 | 2.498 | 0.000 | 2.500 | 0.000 | 2.500 | 0.000 | 2.500 | 0.000 | n/a | n/a | 2.499 | 0.002 | 2.450 | 0.070 | 2.491 | 0.012 |
| 3 | 2.500 | 0.000 | 2.500 | 0.000 | 2.500 | 0.000 | 2.500 | 0.000 | n/a | n/a | 2.499 | 0.001 | 2.462 | 0.054 | 2.500 | 0.000 |
| 6 | 2.497 | 0.004 | 2.500 | 0.000 | 2.500 | 0.000 | 2.500 | 0.000 | 2.495 | 0.000 | 2.500 | 0.001 | 2.463 | 0.041 | 2.494 | 0.009 |
| 9 | 2.500 | 0.000 | 2.500 | 0.000 | 2.500 | 0.000 | 2.500 | 0.000 | 2.500 | 0.000 | 2.500 | 0.000 | 2.484 | 0.022 | 2.496 | 0.006 |
| 12 | 2.500 | 0.000 | 2.500 | 0.000 | 2.500 | 0.000 | 2.500 | 0.000 | n/a | n/a | 2.500 | 0.000 | 2.500 | 0.000 | 2.500 | 0.000 |
| 24 | 2.500 | 0.000 | 2.500 | 0.000 | 2.500 | 0.000 | 2.500 | 0.000 | 2.500 | 0.000 | 2.500 | 0.000 | 2.500 | 0.000 | 2.500 | 0.000 |
| Time | PFDA Control | | PFDA HA | | PFDA EDTA | | PFDA SDS | | PFDA MgCl | | PFDA KNO_3_ | | PFDA Na_3_PO_4_ | | PFDA NaHCO_3_ | |
|  | q_e_ | SD | q_e_ | SD | q_e_ | SD | q_e_ | SD | q_e_ | SD | q_e_ | SD | q_e_ | SD | q_e_ | SD |
| 0 | 0.000 | 0.000 | 0.000 | 0.000 | 0.000 | 0.000 | 0.000 | 0.000 | 2.500 | 0.000 | 0.000 | 0.000 | 0.000 | 0.000 | 0.000 | 0.000 |
| 1 | 2.497 | 0.004 | 2.500 | 0.000 | 2.490 | 0.014 | 2.498 | 0.002 | 2.500 | 0.000 | 2.498 | 0.003 | 2.357 | 0.202 | 2.455 | 0.063 |
| 2 | 2.499 | 0.001 | 2.500 | 0.000 | 2.500 | 0.000 | 2.500 | 0.000 | n/a | n/a | 2.499 | 0.002 | 2.162 | 0.336 | 2.491 | 0.012 |
| 3 | 2.500 | 0.000 | 2.500 | 0.000 | 2.500 | 0.000 | 2.500 | 0.000 | n/a | n/a | 2.499 | 0.001 | 2.290 | 0.204 | 2.500 | 0.000 |
| 6 | 2.500 | 0.000 | 2.500 | 0.000 | 2.500 | 0.000 | 2.500 | 0.000 | 2.500 | 0.000 | 2.500 | 0.001 | 2.316 | 0.152 | 2.500 | 0.000 |
| 9 | 2.500 | 0.000 | 2.500 | 0.000 | 2.500 | 0.000 | 2.500 | 0.000 | 2.500 | 0.000 | 2.500 | 0.000 | 2.362 | 0.150 | 2.500 | 0.000 |
| 12 | 2.500 | 0.000 | 2.500 | 0.000 | 2.500 | 0.000 | 2.500 | 0.000 | n/a | n/a | 2.500 | 0.000 | 2.500 | 0.000 | 2.500 | 0.000 |
| 24 | 2.500 | 0.000 | 2.500 | 0.000 | 2.500 | 0.000 | 2.500 | 0.000 | 2.500 | 0.000 | 2.500 | 0.000 | 2.500 | 0.000 | 2.500 | 0.000 |
| Time | PFUnDA Control | | PFUnDA HA | | PFUnDA EDTA | | PFUnDA SDS | | PFUnDA MgCl | | PFUnDA KNO_3_ | | PFUnDA Na_3_PO_4_ | | PFUnDA NaHCO_3_ | |
|  | q_e_ | SD | q_e_ | SD | q_e_ | SD | q_e_ | SD | q_e_ | SD | q_e_ | SD | q_e_ | SD | q_e_ | SD |
| 0 | 0.000 | 0.000 | 0.000 | 0.000 | 0.000 | 0.000 | 0.000 | 0.000 | 0.000 | 0.000 | 0.000 | 0.000 | 0.000 | 0.000 | 0.000 | 0.000 |
| 1 | 2.480 | 0.029 | 2.480 | 0.029 | 2.241 | 0.121 | 2.491 | 0.012 | 0.688 | 0.055 | 2.493 | 0.010 | 1.337 | 0.005 | 2.497 | 0.005 |
| 2 | 2.494 | 0.009 | 2.500 | 0.000 | 2.425 | 0.101 | 2.493 | 0.010 | n/a | n/a | 2.499 | 0.002 | 1.392 | 0.063 | 2.500 | 0.000 |
| 3 | 2.496 | 0.006 | 2.500 | 0.000 | 2.485 | 0.021 | 2.499 | 0.001 | n/a | n/a | 2.500 | 0.000 | 1.495 | 0.053 | 2.500 | 0.000 |
| 6 | 2.497 | 0.004 | 2.500 | 0.000 | 2.487 | 0.018 | 2.500 | 0.000 | 1.838 | 0.150 | 2.500 | 0.000 | 1.738 | 0.253 | 2.500 | 0.000 |
| 9 | 2.499 | 0.001 | 2.500 | 0.000 | 2.490 | 0.015 | 2.500 | 0.000 | 1.918 | 0.157 | 2.500 | 0.000 | 2.222 | 0.393 | 2.500 | 0.000 |
| 12 | 2.499 | 0.001 | 2.500 | 0.000 | 2.498 | 0.003 | 2.500 | 0.000 | n/a | n/a | 2.500 | 0.000 | 2.500 | 0.000 | 2.500 | 0.000 |
| 24 | 2.500 | 0.000 | 2.500 | 0.000 | 2.497 | 0.001 | 2.500 | 0.000 | 2.029 | 0.167 | 2.500 | 0.000 | 2.500 | 0.000 | 2.500 | 0.000 |

a. PFPeA (C5)

qt (mg/g)

| Time (mins) | PFPeA HA | PFPeA EDTA | PFPeA SDDS | PFPeA MgCl2 | PFPeA KNO3 | PFPeA Na3PO4 | PFPeA NaHCO3 | PFPeA Only |
| --- | --- | --- | --- | --- | --- | --- | --- | --- |
| 0 | 0 | 0 | 0 | 0 | 0 | 0 | 0 | 0 |
| 1 | 3.03736 | 1.4916 | 2.97222 | 1.69312 | 1.55914 | 0.144231 | 1.69154 | 0.855615 |
| 3 | 4.35632 | 3.84244 | 3.50309 | 2.78307 | 2.12366 | 1.25 | 2.60448 | 0.989305 |
| 6 | 4.77011 | 4.0063 | 4.76019 | 3.61111 | 3.73387 | 1.29808 | 3.21642 | 1.25668 |
| 9 | 4.61207 | 4.54412 | 4.86543 | 4.12169 | 3.82527 | 1.5625 | 3.44527 | 1.36364 |
| 24 | 5 | 4.60106 | 4.86108 | 5 | 3.85 | 5 | 3.44527 | 1.63102 |

f. PFPeA (C5)

qt (mg/g)

| Time (mins) | PFPeA HA | PFPeA EDTA | PFPeA MgCl2 | PFPeA SDDS | PFPeA KNO3 | PFPeA Na3PO4 | PFPeA NaHCO3 | PFPeA Only |
| --- | --- | --- | --- | --- | --- | --- | --- | --- |
| 0 | 0 | 0 | 0 | 0 | 0 | 0 | 0 | 0 |
| 1 | 4.91264 | 0.179641 | 3.77513 | 2.3858 | 4.86855 | 4.69406 | 4.51741 | 0.822011 |
| 3 | 5 | 4.82036 | 4.4418 | 5 | 4.87742 | 4.89441 | 4.96891 | 3.27446 |
| 6 | 5 | 5 | 4.75053 | 5 | 4.85242 | 4.78497 | 4.90995 | 4.87683 |
| 9 | 5 | 5 | 4.80132 | 5 | 4.83306 | 4.78846 | 4.96766 | 4.9053 |
| 24 | 5 | 5 | 5 | 5 | 5 | 5 | 5 | 4.93832 |

b. PFOA (C8)

qt (mg/g)

| Time (mins) | PFOA HA | PFOA EDTA | PFOA SDDS | PFOA MgCl2 | PFOA KNO3 | PFOA Na3PO4 | PFOA NaHCO3 | PFOA Only |
| --- | --- | --- | --- | --- | --- | --- | --- | --- |
| 0 | 0 | 0 | 0 | 0 | 0 | 0 | 0 | 0 |
| 1 | 4.56846 | 2.01613 | 4.54156 | 3.02752 | 4.46391 | 0.771277 | 2.28972 | 0.926174 |
| 3 | 5 | 5 | 5 | 4.68463 | 5 | 3.44415 | 5 | 1.45638 |
| 6 | 5 | 5 | 5 | 5 | 5 | 3.75266 | 5 | 1.8255 |
| 9 | 5 | 5 | 5 | 5 | 5 | 5 | 5 | 2.16107 |
| 24 | 5 | 5 | 5 | 5 | 5 | 5 | 5 | 2.84564 |

g. PFOA (C8)

qt (mg/g)

| Time (mins) | PFOA HA | PFOA EDTA | PFOA MgCl2 | PFOA SDDS | PFOA KNO3 | PFOA Na3PO4 | PFOA NaHCO3 | PFOA Only |
| --- | --- | --- | --- | --- | --- | --- | --- | --- |
| 0 | 0 | 0 | 0 | 0 | 0 | 0 | 0 | 0 |
| 1 | 5 | 1.72566 | 4.38188 | 2.44156 | 5 | 5 | 5 | 0.816641 |
| 3 | 5 | 5 | 5 | 5 | 5 | 5 | 5 | 3.25886 |
| 6 | 5 | 5 | 5 | 5 | 5 | 5 | 5 | 4.51233 |
| 9 | 5 | 5 | 5 | 5 | 5 | 5 | 5 | 4.90609 |
| 24 | 5 | 5 | 5 | 5 | 5 | 5 | 5 | 5 |

c. PFNA (C9)

qt (mg/g)

| Time (mins) | PFNA HA | PFNA EDTA | PFNA SDDS | PFNA MgCl2 | PFNA KNO3 | PFNA Na3PO4 | PFNA NaHCO3 | PFNA Only |
| --- | --- | --- | --- | --- | --- | --- | --- | --- |
| 0 | 0 | 0 | 0 | 0 | 0 | 0 | 0 | 0 |
| 1 | 3.89155 | 2.28641 | 0.989305 | 2.53247 | 3.78889 | 0.87069 | 3.01235 | 0.746835 |
| 3 | 5 | 4.88738 | 4.0107 | 4.25081 | 4.96433 | 3.87069 | 4.93568 | 1.87342 |
| 6 | 5 | 4.86408 | 5 | 5 | 5 | 4.6431 | 5 | 2.37975 |
| 9 | 5 | 5 | 5 | 5 | 5 | 5 | 5 | 2.6962 |
| 24 | 5 | 5 | 5 | 5 | 5 | 5 | 5 | 3.67722 |

h. (PFNA C9)

qt (mg/g)

| Time (mins) | PFNA HA | PFNA EDTA | PFNA MgCl2 | PFNA SDDS | PFNA KNO3 | PFNA Na3PO4 | PFNA NaHCO3 | PFNA Only |
| --- | --- | --- | --- | --- | --- | --- | --- | --- |
| 0 | 0 | 0 | 0 | 0 | 0 | 0 | 0 | 0 |
| 1 | 5 | 1.09123 | 0.219968 | 3.18182 | 5 | 5 | 5 | 1 |
| 3 | 5 | 5 | 4.78003 | 5 | 5 | 5 | 5 | 3.40972 |
| 6 | 5 | 5 | 5 | 5 | 5 | 5 | 5 | 4.48681 |
| 9 | 5 | 5 | 5 | 5 | 5 | 5 | 5 | 4.82708 |
| 24 | 5 | 5 | 5 | 5 | 5 | 5 | 5 | 4.96764 |

d. PFDA (C10)

qt (mg/g)

| Time (mins) | PFDA HA | PFDA EDTA | PFDA SDDS | PFDA MgCl2 | PFDA KNO3 | PFDA Na3PO4 | PFDA NaHCO3 | PFDA Only |
| --- | --- | --- | --- | --- | --- | --- | --- | --- |
| 0 | 0 | 0 | 0 | 0 | 0 | 0 | 0 | 0 |
| 1 | 0.79096 | 2.6513 | 2.93438 | 1.11446 | 2.82908 | 1.36364 | 3.21392 | 0.701262 |
| 3 | 3.74576 | 4.10086 | 3.7 | 3.01506 | 3.31888 | 3.94192 | 4.31701 | 2.99439 |
| 6 | 4.70339 | 4.16859 | 4.96063 | 3.91867 | 4.51786 | 4.52525 | 4.91959 | 4.38219 |
| 9 | 5 | 4.47839 | 5 | 4.17169 | 4.66582 | 4.94318 | 5 | 4.20056 |
| 24 | 5 | 5 | 5 | 4.93946 | 5 | 5 | 5 | 5 |

i. PFDA (C10)

qt (mg/g)

| Time (mins) | PFDA HA | PFDA EDTA | PFDA MgCl2 | PFDA SDDS | PFDA KNO3 | PFDA Na3PO4 | PFDA NaHCO3 | PFDA Only |
| --- | --- | --- | --- | --- | --- | --- | --- | --- |
| 0 | 0 | 0 | 0 | 0 | 0 | 0 | 0 | 0 |
| 1 | 4.37006 | 0.337423 | 3.38253 | 0.321637 | 4.65051 | 5 | 5 | 1.38072 |
| 3 | 4.86751 | 4.85307 | 3.72289 | 2.74854 | 5 | 5 | 5 | 4.50245 |
| 6 | 5 | 5 | 5 | 4.59357 | 5 | 5 | 5 | 5 |
| 9 | 5 | 5 | 5 | 5 | 5 | 5 | 5 | 5 |
| 24 | 5 | 5 | 5 | 5 | 5 | 5 | 5 | 5 |

e. PFUnDA (C11)

qt (mg/g)

| Time (mins) | PFUnDA HA | PFUnDA EDTA | PFUnDA SDDS | PFUnDA KNO3 | PFUnDA MgCl2 | PFUnDA Na3PO4 | PFUnDA NaHCO3 | PFUnDA Only |
| --- | --- | --- | --- | --- | --- | --- | --- | --- |
| 0 | 0 | 0 | 0 | 0 | 0 | 0 | 0 | 0 |
| 1 | 1.66 | 0.228758 | 0.219251 | 0.756303 | 0.832444 | 2.17376 | 0.0462428 | 0.958848 |
| 3 | 2.3 | 1.40523 | 2.02139 | 2.10504 | 1.7556 | 2.56738 | 0.421965 | 3.04733 |
| 6 | 3.644 | 3.0915 | 2.2246 | 2.37395 | 1.82497 | 2.56738 | 0.682081 | 3.53498 |
| 9 | 4.11 | 3.0915 | 2.73797 | 2.56303 | 2.32124 | 3.76596 | 0.849711 | 3.89506 |
| 24 | 4.278 | 4.48693 | 3.8877 | 3.81513 | 3.20171 | 4.38652 | 2.68208 | 4.80226 |

j. PFUnDA (C11)

qt (mg/g)

| Time (mins) | PFUnDA HA | PFUnDA EDTA | PFUnDA SDDS | PFUnDA KNO3 | PFUnDA MgCl2 | PFUnDA NaPO4 | PFUnDA NaHCO3 | PFUnDA Only |
| --- | --- | --- | --- | --- | --- | --- | --- | --- |
| 0 | 0 | 0 | 0 | 0 | 0 | 0 | 0 | 0 |
| 1 | 4.18356 | 1.17801 | 2.87253 | 2.98276 | 3.58566 | 2.52187 | 4.52368 | 2.07246 |
| 3 | 4.63671 | 2.65707 | 4.57451 | 4.75948 | 3.94422 | 4.56268 | 4.83565 | 4.21498 |
| 6 | 4.84799 | 4.54647 | 4.6167 | 4.83362 | 4.86554 | 4.88484 | 4.91017 | 4.68792 |
| 9 | 4.63576 | 4.67997 | 4.71724 | 4.8569 | 4.86853 | 4.88776 | 4.93196 | 4.91401 |
| 24 | 4.66635 | 4.90707 | 4.82765 | 4.88448 | 4.87251 | 4.88921 | 4.95822 | 4.9507 |

**Fig. S2.** Batch adsorption kinetics of five PFAS in the presence of seven co-occurring ions (colour coded as follows: PFAS-HA (green), PFAS-EDTA (red), PFAS-SDS (grey), PFAS-MgCl_2_ (orange), PFAS-KNO_3_ (yellow), PFAS-Na_3_PO_4_ (purple) and PFAS- NaHCO₃ (blue) expressed as adsorbed concentration (q_t_, mg/g) at a given time (t, h) on GAC (a-e) and AER (f-j) with C_o_ 10 mg/L. Constant parameters include pH 3.7 ± 0.2, Temperature 28 ± 1 °C and agitation speed 150 RPM.

a. PFPeA (C5)

qt (mg/g)

| Time (mins) | PFPeA HA | PFPeA EDTA | PFPeA SDDS | PFPeA MgCl2 | PFPeA KNO3 | PFPeA Na3PO4 | PFPeA NaHCO3 | PFPeA Only |
| --- | --- | --- | --- | --- | --- | --- | --- | --- |
| 0 | 0 | 0 | 0 | 0 | 0 | 0 | 0 | 0 |
| 1 | 3.125 | 3.73134 | 3.7725 | 3.53972 | 1.04938 | 1.09589 | 1.01449 | 0.631579 |
| 3 | 5.47059 | 4.8694 | 4.09125 | 5.84229 | 1.60494 | 1.40411 | 2.21014 | 0.789474 |
| 6 | 5.99632 | 5.80597 | 5.865 | 6.6028 | 2.96296 | 1.23288 | 2.93478 | 1.10526 |
| 9 | 6.27206 | 6 | 5.97 | 6.69743 | 4.04321 | 7.5 | 3.11594 | 1.57895 |
| 24 | 7.5 | 6.00746 | 5.97187 | 7.5 | 7.5 | 7.5 | 3.11594 | 2.32895 |

f. PFPeA (C5)

qt (mg/g)

| Time (mins) | PFPeA HA | PFPeA EDTA | PFPeA MgCl2 | PFPeA SDDS | PFPeA KNO3 | PFPeA Na3PO4 | PFPeA NaHCO3 | PFPeA Only |
| --- | --- | --- | --- | --- | --- | --- | --- | --- |
| 0 | 0 | 0 | 0 | 0 | 0 | 0 | 0 | 0 |
| 1 | 7.5 | 7.07836 | 0.328786 | 7.41862 | 7.16505 | 6.83848 | 7.46779 | 2.26644 |
| 3 | 7.5 | 7.5 | 6.87967 | 7.5 | 7.19647 | 7.12879 | 7.5 | 5.56228 |
| 6 | 7.5 | 7.5 | 7.24486 | 7.5 | 7.2644 | 7.31081 | 7.5 | 7.31133 |
| 9 | 7.5 | 7.5 | 7.0514 | 7.5 | 7.05628 | 7.31419 | 7.5 | 7.35856 |
| 24 | 7.5 | 7.5 | 7.5 | 7.5 | 7.5 | 7.5 | 7.5 | 7.36117 |

b. PFOA (C8)

qt (mg/g)

| Time (mins) | PFOA HA | PFOA EDTA | PFOA SDDS | PFOA MgCl2 | PFOA KNO3 | PFOA Na3PO4 | PFOA NaHCO3 | PFOA Only |
| --- | --- | --- | --- | --- | --- | --- | --- | --- |
| 0 | 0 | 0 | 0 | 0 | 0 | 0 | 0 | 0 |
| 1 | 4.48073 | 6.7236 | 5.89839 | 5 | 5.09398 | 0.781759 | 3.43902 | 0.58 |
| 3 | 7.5 | 7.5 | 7.5 | 5.29923 | 7.5 | 3.56678 | 6.16829 | 1.68 |
| 6 | 7.5 | 7.5 | 7.5 | 7.5 | 7.5 | 6.04886 | 7.5 | 2.16 |
| 9 | 7.5 | 7.5 | 7.5 | 7.5 | 7.5 | 7.5 | 7.5 | 2.56 |
| 24 | 7.5 | 7.5 | 7.5 | 7.5 | 7.5 | 7.5 | 7.5 | 4.08 |

g. PFOA (C8)

qt (mg/g)

| Time (mins) | PFOA HA | PFOA EDTA | PFOA MgCl2 | PFOA SDDS | PFOA Na3PO4 | PFOA NaHCO3 | PFOA Only | PFOA KNO3 |
| --- | --- | --- | --- | --- | --- | --- | --- | --- |
| 0 | 0 | 0 | 0 | 0 | 0 | 0 | 0 | 0 |
| 1 | 7.5 | 7.5 | 7.5 | 7.5 | 7.5 | 7.5 | 2.46124 | 7.5 |
| 3 | 7.5 | 7.5 | 7.5 | 7.5 | 7.5 | 7.5 | 5.26163 | 7.5 |
| 6 | 7.5 | 7.5 | 7.5 | 7.5 | 7.5 | 7.5 | 6.86143 | 7.5 |
| 9 | 7.5 | 7.5 | 7.5 | 7.5 | 7.5 | 7.5 | 7.31347 | 7.5 |
| 24 | 7.5 | 7.5 | 7.5 | 7.5 | 7.5 | 7.5 | 7.5 | 7.5 |

c. (PFNA C9)

qt (mg/g)

| Time (mins) | PFNA HA | PFNA EDTA | PFNA SDDS | PFNA MgCl2 | PFNA KNO3 | PFNA Na3PO4 | PFNA NaHCO3 | PFNA Only |
| --- | --- | --- | --- | --- | --- | --- | --- | --- |
| 0 | 0 | 0 | 0 | 0 | 0 | 0 | 0 | 0 |
| 1 | 2.98077 | 5.45332 | 5.45332 | 4.64844 | 4.9537 | 2.69591 | 4.64844 | 0.638889 |
| 3 | 7.49577 | 7.5 | 7.5 | 7.5 | 7.5 | 6.25298 | 7.5 | 2.32407 |
| 6 | 7.5 | 7.5 | 7.5 | 7.5 | 7.43576 | 7.5 | 7.5 | 3.09259 |
| 9 | 7.5 | 7.5 | 7.5 | 7.5 | 7.5 | 7.5 | 7.5 | 3.53704 |
| 24 | 7.5 | 7.5 | 7.5 | 7.5 | 7.5 | 7.5 | 7.5 | 5.2963 |

h. (PFNA C9)

qt (mg/g)

| Time (mins) | PFNA HA | PFNA EDTA | PFNA MgCl2 | PFNA SDDS | PFNA Na3PO4 | PFNA NaHCO3 | PFNA Only | PFNA KNO3 |
| --- | --- | --- | --- | --- | --- | --- | --- | --- |
| 0 | 0 | 0 | 0 | 0 | 0 | 0 | 0 | 0 |
| 1 | 7.5 | 7.5 | 7.5 | 7.5 | 7.5 | 7.5 | 2.62457 | 7.5 |
| 3 | 7.5 | 7.5 | 7.5 | 7.5 | 7.5 | 7.5 | 5.20568 | 7.5 |
| 6 | 7.5 | 7.5 | 7.5 | 7.5 | 7.5 | 7.5 | 6.76477 | 7.5 |
| 9 | 7.5 | 7.5 | 7.5 | 7.5 | 7.5 | 7.5 | 7.24363 | 7.5 |
| 24 | 7.5 | 7.5 | 7.5 | 7.5 | 7.5 | 7.5 | 7.45724 | 7.5 |

d. PFDA (C10)

qt (mg/g)

| Time (mins) | PFDA HA | PFDA EDTA | PFDA SDDS | PFDA MgCl2 | PFDA KNO3 | PFDA Na3PO4 | PFDA NaHCO3 | PFDA Only |
| --- | --- | --- | --- | --- | --- | --- | --- | --- |
| 0 | 0 | 0 | 0 | 0 | 0 | 0 | 0 | 0 |
| 1 | 4.07216 | 2.475 | 4.87092 | 2.82178 | 5.37572 | 3.20856 | 4.24749 | 0.627615 |
| 3 | 6.75258 | 5.4075 | 6.50136 | 7.14765 | 5.51879 | 6.45321 | 5.8907 | 3.58787 |
| 6 | 6.99485 | 5.565 | 7.33777 | 7.34146 | 5.88295 | 6.96257 | 5.8907 | 4.9477 |
| 9 | 7.38196 | 5.89875 | 7.5 | 7.41126 | 6.46821 | 7.5 | 7.2995 | 5.61715 |
| 24 | 7.5 | 7.5 | 7.5 | 7.5 | 7.5 | 7.5 | 7.5 | 7.5 |

i. PFDA (C10)

qt (mg/g)

| Time (mins) | PFDA HA | PFDA MgCl2 | PFDA SDDS | PFDA EDTA | PFDA Na3PO4 | PFDA NaHCO3 | PFDA Only | PFDA KNO3 |
| --- | --- | --- | --- | --- | --- | --- | --- | --- |
| 0 | 0 | 0 | 0 | 0 | 0 | 0 | 0 | 0 |
| 1 | 7.25865 | 5.96287 | 7.40625 | 7.23337 | 6.5615 | 7.5 | 3.76913 | 7.18092 |
| 3 | 7.5 | 7.5 | 7.5 | 7.4682 | 7.5 | 7.5 | 6.13202 | 7.19306 |
| 6 | 7.5 | 7.5 | 7.5 | 7.5 | 7.5 | 7.5 | 7.5 | 7.5 |
| 9 | 7.5 | 7.5 | 7.5 | 7.5 | 7.5 | 7.5 | 7.5 | 7.5 |
| 24 | 7.5 | 7.5 | 7.5 | 7.5 | 7.5 | 7.5 | 7.5 | 7.5 |

e. PFUnDA (C11)

qt (mg/g)

| Time (mins) | PFUnDA HA | PFUnDA EDTA | PFUnDA MgCl2 | PFUnDA SDDS | PFUnDA KNO3 | PFUnDA Na3PO4 | PFUnDA NaHCO3 | PFUnDA Only |
| --- | --- | --- | --- | --- | --- | --- | --- | --- |
| 0 | 0 | 0 | 0 | 0 | 0 | 0 | 0 | 0 |
| 1 | 2.49 | 0.3 | 1.31198 | 0.350365 | 1.69811 | 1.1217 | 1.62704 | 3.20248 |
| 3 | 3.45 | 3.3 | 2.07645 | 1.34672 | 3.99057 | 1.41862 | 2.12853 | 5.23347 |
| 6 | 5.466 | 4.98375 | 2.61364 | 3.16423 | 4.67453 | 3.02419 | 2.17311 | 5.93388 |
| 9 | 6.165 | 4.98375 | 3.27479 | 3.43796 | 4.86792 | 5.69648 | 2.69688 | 6.29752 |
| 24 | 6.417 | 6.49875 | 5.41322 | 5.34307 | 6.48113 | 7.04142 | 6.45579 | 6.83221 |

j. PFUnDA (C11)

qt (mg/g)

| Time (mins) | PFUnDA HA | PFUnDA EDTA | PFUnDA SDDS | PFUnDA KNO3 | PFUnDA MgCl2 | PFUnDA NaPO4 | PFUnDA Only | PFUnDA NaHCO3 |
| --- | --- | --- | --- | --- | --- | --- | --- | --- |
| 0 | 0 | 0 | 0 | 0 | 0 | 0 | 0 | 0 |
| 1 | 6.45243 | 6.82712 | 6.24956 | 6.30914 | 6.34057 | 4.67375 | 3.53306 | 6.69874 |
| 3 | 6.99447 | 6.76453 | 6.92482 | 6.34946 | 6.65735 | 6.91935 | 5.49793 | 7.08432 |
| 6 | 7.21792 | 7.11215 | 6.98488 | 7.00403 | 7.24082 | 7.05902 | 6.8312 | 7.21694 |
| 9 | 7.35066 | 7.31557 | 7.13301 | 7.24866 | 7.25083 | 7.34714 | 7.23347 | 7.37407 |
| 24 | 7.31195 | 7.33793 | 7.24244 | 7.26344 | 7.351 | 7.35924 | 7.42227 | 7.38744 |

**Fig. S3.** Batch adsorption kinetics of five PFAS in the presence of seven co-occurring ions (colour coded as follows: PFAS-HA (green), PFAS-EDTA (red), PFAS-SDS (grey), PFAS-MgCl_2_ (orange), PFAS-KNO_3_ (yellow), PFAS-Na_3_PO_4_ (purple) and PFAS- NaHCO₃ (blue) expressed as adsorbed concentration (q_t_, mg/g) at a given time (t, h) on GAC (a-e) and AER (f-j) with C_o_ 15 mg/L. Constant parameters include pH 3.7 ± 0.2, Temperature 28 ± 1 °C and agitation speed 150 RPM.

a. PFPeA (C5)

qt (mg/g)

| Time (mins) | PFPeA HA | PFPeA EDTA |
| --- | --- | --- |
| 0 | 0 | 0 |
| 1 | 5.86538 | 0.651659 |
| 3 | 8.42308 | 4.02844 |
| 6 | 8.47115 | 3.73223 |
| 9 | 8.79327 | 7.35782 |
| 24 | 12.5 | 7.381 |

| Time (mins) | PFPeA SDDS |
| --- | --- |
| 0 | 0 |
| 1 | 2.43902 |
| 3 | 2.7439 |
| 6 | 5.36585 |
| 9 | 8.83537 |
| 24 | 8.845 |

| Time (mins) | PFPeA MgCl2 | PFPeA KNO3 | PFPeA Na3PO4 | PFPeA NaHCO3 | PFPeA Only |
| --- | --- | --- | --- | --- | --- |
| 0 | 0 | 0 | 0 | 0 | 0 |
| 1 | 3.73563 | 2.39899 | 1.0101 | 0.10917 | 1.16279 |
| 3 | 7.91188 | 2.23064 | 4.0404 | 0.927948 | 2.32558 |
| 6 | 8.40038 | 5.26094 | 5.30303 | 1.4738 | 2.76163 |
| 9 | 10.1628 | 6.43939 | 7.92298 | 1.37665 | 2.68895 |
| 24 | 12.5 | 12.5 | 12.5 | 1.88679 | 4.14244 |

f. PFPeA (C5)

qt (mg/g)

| Time (mins) | PFPeA HA | PFPeA EDTA |
| --- | --- | --- |
| 0 | 0 | 0 |
| 1 | 12.3149 | 10.1126 |
| 3 | 12.5 | 12.5 |
| 6 | 12.5 | 12.5 |
| 9 | 12.5 | 12.2245 |
| 24 | 12.5 | 12.5 |

| Time (mins) | PFPeA MgCl2 | PFPeA SDDS |
| --- | --- | --- |
| 0 | 0 | 0 |
| 1 | 11.796 | 12.1854 |
| 3 | 11.7577 | 12.5 |
| 6 | 11.978 | 12.5 |
| 9 | 11.5948 | 12.5 |
| 24 | 12.5 | 12.5 |

| Time (mins) | PFPeA KNO3 | PFPeA Na3PO4 | PFPeA NaHCO3 | PFPeA Only |
| --- | --- | --- | --- | --- |
| 0 | 0 | 0 | 0 | 0 |
| 1 | 11.2396 | 11.8805 | 10.0819 | 3.56922 |
| 3 | 12.2661 | 12.2122 | 11.6703 | 6.42769 |
| 6 | 12.0583 | 12.1261 | 12.4643 | 7.97281 |
| 9 | 11.6562 | 12.0082 | 12.4318 | 11.1218 |
| 24 | 12.5 | 12.5 | 12.5 | 12.4578 |

b. PFOA (C8)

qt (mg/g)

| Time (mins) | PFOA HA |
| --- | --- |
| 0 | 0 |
| 1 | 8.575 |
| 3 | 12.5 |
| 6 | 12.5 |
| 9 | 12.5 |
| 24 | 12.5 |

| Time (mins) | PFOA EDTA | PFOA SDDS |
| --- | --- | --- |
| 0 | 0 | 0 |
| 1 | 5.7947 | 9.54093 |
| 3 | 12.4011 | 12.5 |
| 6 | 12.5 | 12.5 |
| 9 | 12.5 | 12.5 |
| 24 | 12.5 | 12.5 |

| Time (mins) | PFOA MgCl2 |
| --- | --- |
| 0 | 0 |
| 1 | 9.64912 |
| 3 | 12.5 |
| 6 | 12.5 |
| 9 | 12.5 |
| 24 | 12.5 |

| Time (mins) | PFOA KNO3 | PFOA Na3PO4 | PFOA NaHCO3 | PFOA Only |
| --- | --- | --- | --- | --- |
| 0 | 0 | 0 | 0 | 0 |
| 1 | 0.122309 | 2.8972 | 7.29638 | 1.82563 |
| 3 | 9.83366 | 7.95561 | 7.74887 | 1.99329 |
| 6 | 12.5 | 8.8785 | 11.2443 | 2.86885 |
| 9 | 12.5 | 11.2383 | 11.5865 | 2.81297 |
| 24 | 12.5 | 12.5 | 12.5 | 4.73174 |

g. PFOA (C8)

qt (mg/g)

| Time (mins) | PFOA HA | PFOA MgCl2 |
| --- | --- | --- |
| 0 | 0 | 0 |
| 1 | 8.575 | 12.5 |
| 3 | 12.5 | 12.5 |
| 6 | 12.5 | 12.5 |
| 9 | 12.5 | 12.5 |
| 24 | 12.5 | 12.5 |

| Time (mins) | PFOA SDDS | PFOA KNO3 | PFOA Na3PO4 | PFOA NaHCO3 | PFOA Only | PFOA EDTA |
| --- | --- | --- | --- | --- | --- | --- |
| 0 | 0 | 0 | 0 | 0 | 0 | 0 |
| 1 | 12.5 | 12.5 | 12.5 | 12.5 | 4.29004 | 10.2442 |
| 3 | 12.5 | 12.5 | 12.5 | 12.5 | 7.57066 | 12.5 |
| 6 | 12.5 | 12.5 | 12.5 | 12.5 | 10.7958 | 12.5 |
| 9 | 12.5 | 12.5 | 12.5 | 12.5 | 12.039 | 12.5 |
| 24 | 12.5 | 12.5 | 12.5 | 12.5 | 12.5 | 12.5 |

c. (PFNA C9)

qt (mg/g)

| Time (mins) | PFNA HA | PFNA EDTA | PFNA SDDS | PFNA MgCl2 | PFNA KNO3 | PFNA Na3PO4 | PFNA NaHCO3 | PFNA Only |
| --- | --- | --- | --- | --- | --- | --- | --- | --- |
| 0 | 0 | 0 | 0 | 0 | 0 | 0 | 0 | 0 |
| 1 | 8.63575 | 8.77544 | 9.49074 | 9.48383 | 1.48305 | 3.86364 | 4.87589 | 0.688073 |
| 3 | 12.5 | 12.1911 | 12.5 | 12.5 | 10.0106 | 6.85606 | 9.89362 | 2.35092 |
| 6 | 12.5 | 12.5 | 12.5 | 12.5 | 12.5 | 10.5455 | 12.4301 | 3.17278 |
| 9 | 12.5 | 12.2456 | 12.5 | 12.5 | 12.5 | 12.2189 | 12.5 | 4.01376 |
| 24 | 12.5 | 12.5 | 12.5 | 12.5 | 12.5 | 12.5 | 12.5 | 6.6896 |

h. (PFNA C9)

qt (mg/g)

| Time (mins) | PFNA HA | PFNA EDTA | PFNA MgCl2 | PFNA SDDS | PFNA KNO3 | PFNA Na3PO4 | PFNA NaHCO3 | PFNA Only |
| --- | --- | --- | --- | --- | --- | --- | --- | --- |
| 0 | 0 | 0 | 0 | 0 | 0 | 0 | 0 | 0 |
| 1 | 12.5 | 9.28416 | 12.5 | 12.5 | 12.5 | 12.5 | 11.0957 | 2.49695 |
| 3 | 12.5 | 12.5 | 12.5 | 12.5 | 12.5 | 12.5 | 12.5 | 7.79537 |
| 6 | 12.5 | 12.5 | 12.5 | 12.5 | 12.5 | 12.5 | 12.5 | 10.8252 |
| 9 | 12.5 | 12.5 | 12.5 | 12.5 | 12.5 | 12.5 | 12.5 | 11.9945 |
| 24 | 12.5 | 12.5 | 12.5 | 12.5 | 12.5 | 12.5 | 12.5 | 12.4055 |

d.PFDA (C10)

qt (mg/g)

| Time (mins) | PFDA HA | PFDA EDTA | PFDA MgCl2 | PFDA SDDS | PFDA KNO3 | PFDA Na3PO4 | PFDA NaHCO3 | PFDA Only |
| --- | --- | --- | --- | --- | --- | --- | --- | --- |
| 0 | 0 | 0 | 0 | 0 | 0 | 0 | 0 | 0 |
| 1 | 5.51724 | 8.02956 | 8.27569 | 3.94585 | 1.02273 | 5.77091 | 4.86111 | 4.32036 |
| 3 | 11.9655 | 10.0123 | 12.2653 | 11.5841 | 9.125 | 7.70906 | 10.2604 | 6.77937 |
| 6 | 12.0172 | 10.936 | 12.5 | 12.1561 | 11.1307 | 11.385 | 12.0174 | 7.76981 |
| 9 | 12.2353 | 11.7118 | 12.5 | 12.5 | 11.767 | 11.9882 | 12.1499 | 9.20423 |
| 24 | 12.5 | 12.5 | 12.5 | 12.5 | 12.5 | 12.5 | 12.5 | 12.2029 |

i. PFDA (C10)

qt (mg/g)

| Time (mins) | PFDA HA | PFDA EDTA | PFDA MgCl2 | PFDA SDDS | PFDA KNO3 | PFDA Na3PO4 | PFDA NaHCO3 | PFDA Only |
| --- | --- | --- | --- | --- | --- | --- | --- | --- |
| 0 | 0 | 0 | 0 | 0 | 0 | 0 | 0 | 0 |
| 1 | 12.4913 | 7.73399 | 11.3241 | 12.5 | 12.5 | 11.9091 | 9.40972 | 4.23515 |
| 3 | 11.7457 | 12.5 | 12.0934 | 12.5 | 12.5 | 12.5 | 12.5 | 9.26043 |
| 6 | 12.5 | 12.5 | 12.5 | 12.5 | 12.5 | 12.5 | 12.5 | 12.5 |
| 9 | 12.5 | 12.5 | 12.5 | 12.5 | 12.5 | 12.5 | 12.5 | 12.5 |
| 24 | 12.5 | 12.5 | 12.5 | 12.5 | 12.5 | 12.5 | 12.5 | 12.5 |

e. PFUnDA (C11)

qt (mg/g)

| Time (mins) | PFUnDA HA | PFUnDA EDTA | PFUnDA MgCl2 | PFUnDA SDDS | PFUnDA KNO3 | PFUnDA Na3PO4 | PFUnDA NaHCO3 | PFUnDA Only |
| --- | --- | --- | --- | --- | --- | --- | --- | --- |
| 0 | 0 | 0 | 0 | 0 | 0 | 0 | 0 | 0 |
| 1 | 7.07547 | 5.65278 | 1.53093 | 1.42045 | 1.63043 | 4.97788 | 2.08333 | 5.32358 |
| 3 | 8.9033 | 7.91667 | 5.53977 | 2.00758 | 5.93297 | 8.32965 | 7.72059 | 8.19592 |
| 6 | 9.22563 | 9.34028 | 6.23422 | 2.63258 | 7.02899 | 9.8396 | 8.01821 | 9.26862 |
| 9 | 11.305 | 9.5 | 7.21275 | 8.27652 | 7.22826 | 11.3385 | 8.61345 | 10.031 |
| 24 | 11.4072 | 10.8542 | 7.44949 | 11.4223 | 11.2862 | 12.1062 | 10.9769 | 11.5248 |

j. PFUnDA (C11)

qt (mg/g)

| Time (mins) | PFUnDA HA | PFUnDA EDTA | PFUnDA SDDS | PFUnDA KNO3 |
| --- | --- | --- | --- | --- |
| 0 | 0 | 0 | 0 | 0 |
| 1 | 10.3691 | 7.27273 | 11.238 | 11.9534 |
| 3 | 11.0009 | 11.4905 | 11.5091 | 11.9793 |
| 6 | 12.0258 | 12.0644 | 11.3076 | 12.0017 |
| 9 | 12.0993 | 12.1667 | 11.9218 | 12.2793 |
| 24 | 12.0588 | 12.2424 | 12.1257 | 12.281 |

| Time (mins) | PFUnDA MgCl2 |
| --- | --- |
| 0 | 0 |
| 1 | 9.13669 |
| 3 | 10.9119 |
| 6 | 12.1277 |
| 9 | 12.1763 |
|  | 12.2158 |

| Time (mins) | PFUnDA NaPO4 | PFUnDA NaHCO3 | PFUnDA Only |
| --- | --- | --- | --- |
| 0 | 0 | 0 | 0 |
| 1 | 8.85279 | 8.78852 | 3.43605 |
| 3 | 9.73143 | 11.8137 | 7.97674 |
| 6 | 11.4804 | 11.9293 | 10.9942 |
| 9 | 12.0623 | 12.2777 | 11.8983 |
| 24 | 12.2298 | 12.2882 | 12.3547 |

**Fig. S4.** Batch adsorption kinetics of five PFAS in the presence of seven co-occurring ions (colour coded as follows: PFAS-HA (green), PFAS-EDTA (red), PFAS-SDS (grey), PFAS-MgCl_2_ (orange), PFAS-KNO_3_ (yellow), PFAS-Na_3_PO_4_ (purple) and PFAS- NaHCO₃ (blue) expressed as adsorbed concentration (q_t_, mg/g) at a given time (t, h) on GAC (a-e) and AER (f-j) with C_o_ 25 mg/L. Constant parameters include pH 3.7 ± 0.2, Temperature 28 ± 1 °C and agitation speed 150 RPM.

**Table S4.** PFAS LC-MS Acquisition Method (Fatima et al., 2026).

| LCMS Instrument | Water™ Acquity Ultra Performance Liquid Chromatography system (UPLC), equipped with a QTRAP5500 mass spectrometer (Sciex, USA). |
| --- | --- |
| Analytical Column | Acquity HSS T3 (50 mm X 2.1 mm id., 1.8 µm particle size) with VanGuard Pre-Column (Acquity HSS T3 1.8 μm VanGuard) |
| Solvent Delay Column | Raptor Polar X LC column |
| Column Temperature | 35 |
| Injection Volume | 1µL |
| LC Flow Rate | 0.3 mL/min |
| Mobile Phase A | 10 mM Ammonium Acetate (NH_4_OAc); (192.5 mg NH_4_OAcC in 250 mL MilliQ-H_2_O) |
| Mobile Phase B | 10 mM Ammonium Acetate (NH_4_OAc) Methanol: Acetonitrile (80:20); (192.5 mg NH_4_OAc in 200 mL Methanol and 50 mL Acetonitrile) |
| Detection | ESI MS/MS |
| Ionization | Heated electrospray ionization in negative mode |
| Gradient Conditions | Time (min) \| Solvent line A (%) \| Solvent line B (%); 0 \| 60 \| 40; 0-8 \| 10 \| 90; 8-10 \| 10 \| 90; 10-11 \| 90-40 \| 10-60; 11-12 (reconditioning) \| 60 \| 40 |
| Run/Acquisition Cycle Time | 20 min |
| Calibration curve | Instrument response for each PFAS was calibrated against 10 solutions of PFAS standard mixture as concentrations: 0.01, 0.02, 0.05, 0.1, 0.2, 0.3, 0.4, 0.5, 0.6 and 0.7 mg/L in 300 µL |
| Conditions for ESI-MSMS | Ion spray voltage -4500 V, ion source temperature 600 ℃, curtain gas 20 psi, nebulizer gas (GS1) 20 psi, auxiliary gas (GS2) 20 psi |

**Table S5.** Mass Spectrometry (MS) optimization parameters and Multiple Reaction Monitoring (MRM) transitions for target analytes (Fatima et al., 2026).

| No. | Code |  | RT | Precursor ion (*m/z*) | DP | EP | CXP | Product 1  (Q) | CE | Product 2  (ID) | CE |
| --- | --- | --- | --- | --- | --- | --- | --- | --- | --- | --- | --- |
| 5 | PFPeA | 263 | 2.0 | 262.9 | -50 | -9 | -27 | 218.7 | -13 | 130.7 | -25 |
| 8 | PFOA | 413 | 5.0 | 413.2 | -50 | -10 | -13 | 369 | -15 | 169 | -25 |
| 9 | PFNA | 463 | 5.8 | 463.2 | -36 | -4 | -36 | 419 | -16 | 219 | -24 |
| 10 | PFDA | 513 | 6.5 | 512.9 | -50 | -6 | -13 | 468.9 | -15 | 218.5 | -25 |
| 12 | PFUnDA | 563 | 7.1 | 562.9 | -75 | -10 | -17 | 518.9 | -14 | 269 | -25 |

**Determination of Limits of Detection (LoDs) and Quantification (LoQs)**

Excellent linearity (R^2^ >97) of the quantification method (LC-MS/MS, Section 2.6.1) was obtained over the established measurements ranges of 0.01-0.7 mg/L depending on the compound. The limit of detection (LoD) and limit of quantification (LoQ) were determined through signal to noise ratio data response ratios of 3 and 10, respectively, and provided in LoD ranged from 0.01-0.1 mg/L and LoQ ranged from 0.2-0.4 mg/L depending on the compounds allowing for quantification of each PFAS even at low concentrations (Fatima et al., 2026).

**Table S6**. LoDs and LoQs for the five targeted PFAS measure through signal to noise ratio (Fatima et al., 2026).

| PFAS | LoD (mg/L) | LoQ (mg/L) | R^2^ |
| --- | --- | --- | --- |
| PFPeA | 0.01 | 0.2 | 0.97 |
| PFOA | 0.05 | 0.2 | 0.99 |
| PFNA | 0.05 | 0.2 | 0.99 |
| PFDA | 0.05 | 0.4 | 0.99 |
| PFUnDA | 0.1 | 0.3 | 0.98 |

**Table S7.** Experimentally measure and Pseudo-second order model calculated adsorption capacity (q_e_, mg/g) of PFAS on GAC and AER in the presence of co-occurring organic (EDTA, SDDA, HA) and inorganic ions (MgCl₂, KNO₃, Na₃PO₄, and NaHCO₃). χ² (%) values corresponding to model goodness-of-fit. Constant parameters include C_o_ 10 mg/L, pH 3.7 ± 0.2, Temperature 28 ± 1 °C and agitation speed 150 RPM.

| PFAS | AC | | | | AER | | | |
| --- | --- | --- | --- | --- | --- | --- | --- | --- |
|  | Qe | K_2_ | R^2^ | X^2^ % | Qe | K_2_ | R^2^ | X^2^ % |
| PFPeA Control | 1.11 | 0.018 | 0.99 | 2.99 | 5.54 | 0.0014 | 0.95 | 11.51 |
| PFPeA HA | 5.06 | 0.0073 | 0.99 | 1.18 | 5 | 0.6724 | 0.99 | 0 |
| PFPeA EDTA | 4.79 | 0.0036 | 0.99 | 3.96 | 8.62 | 0.00015 | 0.2 | 42.02 |
| PFPeA SDS | 4.97 | 0.0068 | 0.99 | 2.21 | 5.08 | 0.0105 | 0.99 | 1.71 |
| PFPeA MgCl | 5.28 | 0.0016 | 0.99 | 5.3 | 5.04 | 0.0113 | 0.99 | 0.82 |
| PFPeA KNO3 | 4.04 | 0.0037 | 0.99 | 4.7 | 5 | 0.0328 | 0.99 | 0.03 |
| PFPeA Na3PO4 | 11.88 | 3.38-05 | 0.089 | 57.91 | 5 | 0.0242 | 0.99 | 0.06 |
| PFPeA NaHCO3 | 3.54 | 0.0075 | 0.99 | 2.82 | 5 | 0.0242 | 0.99 | 0.06 |
| PFOA Control | 2.99 | 0.002 | 0.97 | 4.79 | 5.67 | 0.0012 | 0.95 | 11.87 |
| PFOA HA | 5 | 0.126 | 0.99 | 0 | 5 | 0 | 1 | 0 |
| PFOA EDTA | 5.12 | 0.00013 | 0.99 | 2.15 | 5.15 | 0.00013 | 0.99 | 2.96 |
| PFOA SDS | 5 | 0.12 | 0.99 | 0 | 5.08 | 0.01105 | 0.99 | 1.63 |
| PFOA MgCl | 5.06 | 0.0141 | 0.99 | 1.18 | 5.011 | 0.08444 | 0.99 | 0.22 |
| PFOA KNO3 | 5 | 0.099 | 0.99 | 0 | 5 | 0 | 1 | 0 |
| PFOA Na3PO4 | 5.59 | 0.0012 | 0.94 | 10.55 | 5 | 0 | 1 | 0 |
| PFOA NaHCO3 | 5.09 | 0.00973 | 0.99 | 1.76 | 5 | 0 | 1 | 0 |
| PFNA Control | 2.99 | 0.002 | 0.96 | 23.02 | 5.51 | 0.0015 | 0.97 | 9.95 |
| PFNA HA | 5.02 | 0.041 | 0.99 | 0.44 | 5 | 0 | 1 | 0 |
| PFNA EDTA | 5.1 | 0.0084 | 0.99 | 1.97 | 5.29 | 0.0029 | 0.97 | 5.59 |
| PFNA SDS | 5.38 | 0.0021 | 0.97 | 7.06 | 5.04 | 0.0205 | 0.99 | 0.89 |
| PFNA MgCl | 5.1 | 0.0032 | 0.99 | 2.07 | 5 | 0.00637 | 0.99 | 0 |
| PFNA KNO3 | 5.02 | 0.0349 | 0.99 | 0.52 | 5 | 0 | 1 | 0 |
| PFNA Na3PO4 | 5.46 | 0.00172 | 0.96 | 8.42 | 5 | 0 | 1 | 0 |
| PFNA NaHCO3 | 5.05 | 0.0168 | 0.99 | 1.07 | 5 | 0 | 1 | 0 |
| PFDA Control | 4.99 | 0.001 | 0.94 | 12.44 | 5.41 | 0.001 | 0.97 | 8.75 |
| PFDA HA | 5.52 | 0.0015 | 0.95 | 9.48 | 5.01 | 0.055 | 0.99 | 0.31 |
| PFDA EDTA | 5.1 | 0.0041 | 0.99 | 1.94 | 6.38 | 0.00052 | 0.63 | 21.67 |
| PFDA SDS | 5.11 | 0.0069 | 0.99 | 2.15 | 6.7 | 0.00039 | 0.61 | 25.46 |
| PFDA MgCl | 5.31 | 0.0015 | 0.98 | 5.938 | 5.09 | 0.0083 | 0.99 | 1.83 |
| PFDA KNO3 | 5.13 | 0.004 | 0.99 | 2.69 | 5 | 0.1588 | 0.99 | 0.11 |
| PFDA Na3PO4 | 5.27 | 0.0028 | 0.81 | 5.15 | 5 | 0 | 1 | 0 |
| PFDA NaHCO3 | 5.07 | 0.0112 | 0.99 | 1.39 | 5 | 0 | 1 | 0 |
| PFUnDA Control | 5.2 | 0.001 | 0.97 | 7.65 | 5.18 | 0.0035 | 0.99 | 4.55 |
| PFUnDA HA | 4.52 | 0.00266 | 0.99 | 5.32 | 4.67 | 1.43081 | 0.99 | 0.1 |
| PFUnDA EDTA | 6.89 | 0.0002 | 0.46 | 34.87 | 5.3 | 0.00176 | 0.98 | 7.56 |
| PFUnDA SDS | 5.45 | 0.00032 | 0.56 | 28.62 | 4.88 | 0.01088 | 0.99 | 1.22 |
| PFUnDA MgCl | 3.435 | 0.0002 | 0.96 | 6.85 | 4.94 | 0.00014 | 0.99 | 1.07 |
| PFUnDA KNO3 | 4.14 | 0.00123 | 0.95 | 7.92 | 4.94 | 0.0155 | 0.99 | 1.3 |
| PFUnDA Na3PO4 | 4.588 | 0.0019 | 0.97 | 4.4 | 4.97 | 0.0104 | 0.99 | 1.69 |

**(a)**

**(b)**

**(c)**

**(d)**

**Fig. S5.** Total ion chromatograms (TICs) and extracted ion chromatograms (XICs) for (a) PFAS standards prepared in Milli‑Q water (0.156 µg/mL, 2 µL injection), (b) PFAS standards prepared in MeOH (0.156 µg/mL, 2 µL injection), (c) Milli‑Q blank, and (d) MeOH Blank analysed before and between experimental samples.

**Table S8**. Removal efficiencies of PFAS at varying initial concentrations (5, 10, 15, and 25 mg/L) on GAC and AER. The standard deviation for 5 mg/L concentration is calculated from duplicate experiments.

| Adsorbent | AC | | | | AER | | | |
| --- | --- | --- | --- | --- | --- | --- | --- | --- |
| PFAS C_o_ | 5 | 10 | 15 | 25 | 5 | 10 | 15 | 25 |
| PFPeA | 80 ± 9.14 | 78 | 68 | 90 | 100 ± 0.00 | 99 | 98 | 100 |
| PFOA | 89 ± 2.64 | 57 | 54 | 62 | 100 ± | 100 | 100 | 100 |
| PFNA | 88 ± 8.17 | 74 | 71 | 54 | 100 ± 0.58 | 99 | 99 | 99 |
| PFDA | 72 ± 18.45 | 88 | 87 | 82 | 100 ± 0.00 | 99 | 99 | 99 |
| PFUnDA | 77 ± 1.76 | 91 | 91 | 92 | 99 ± 2.30 | 99 | 99 | 99 |

**Table S9.** Adsorption efficiency percentage (%) along the standard deviation (SD) of duplicates, of five PFAS in the presence of co-occurring ions on GAC and AER.

| Experiment | PFPeA | | PFOA | | PFNA | | PFDA | | PFUnDA | |
| --- | --- | --- | --- | --- | --- | --- | --- | --- | --- | --- |
|  | % | SD | % | SD | % | SD | % | SD | % | SD |
| AER CNT | 96 | 3.82 | 100 | 0.00 | 100 | 0.58 | 100 | 0.00 | 99 | 2.31 |
| AC CNT | 80 | 9.13 | 89 | 2.64 | 88 | 8.17 | 72 | 18.45 | 77 | 1.76 |
| AER HA | 99 | 0.55 | 100 | 0.00 | 100 | 0.00 | 100 | 0.00 | 100 | 0.00 |
| AC HA | 100 | 0.46 | 100 | 0.00 | 100 | 0.83 | 96 | 4.02 | 94 | 5.54 |
| AER EDTA | 99 | 1.06 | 100 | 0.00 | 100 | 0.00 | 100 | 0.00 | 99 | 1.08 |
| AC EDTA | 100 | 0.32 | 99 | 1.61 | 99 | 1.02 | 73 | 3.55 | 83 | 1.78 |
| AER SDS | 100 | 0.29 | 100 | 0.00 | 100 | 0.00 | 100 | 0.00 | 99 | 1.15 |
| AC SDS | 100 | 0.19 | 98 | 4.10 | 94 | 9.43 | 67 | 4.63 | 42 | 1.31 |
| AER MgCl_2_ | 92 | 0.87 | 100 | 0.00 | 100 | 0.00 | 100 | 0.00 | 99 | 1.73 |
| AC MgCl_2_ | 99 | 0.74 | 100 | 0.00 | 99 | 1.94 | 97 | 5.40 | 79 | 5.90 |
| AER KNO_3_ | 95 | 4.13 | 100 | 0.00 | 100 | 0.00 | 100 | 0.00 | 99 | 1.73 |
| AC KNO_3_ | 97 | 0.92 | 100 | 0.68 | 96 | 5.45 | 89 | 5.02 | 83 | 2.64 |
| AER Na_3_PO_4_ | 94 | 4.89 | 100 | 0.00 | 100 | 0.00 | 100 | 0.00 | 100 | 0.58 |
| AC Na_3_PO_4_ | 97 | 2.67 | 100 | 0.00 | 100 | 0.00 | 100 | 0.00 | 100 | 0.00 |
| AER NaHCO_3_ | 96 | 3.43 | 100 | 0.00 | 100 | 0.00 | 100 | 3.18 | 99 | 1.73 |
| AC NaHCO_3_ | 95 | 5.80 | 100 | 0.03 | 99 | 1.18 | 98 | 3.76 | 77 | 1.00 |

**Table S10.** Desorption efficiency (%) percentage along the standard deviation (SD) of duplicates, of five PFAS in the presence of co-occurring ions on GAC and AER.

| Experiment | PFPeA | | PFOA | | PFNA | | PFDA | | PFUnDA | |
| --- | --- | --- | --- | --- | --- | --- | --- | --- | --- | --- |
|  | % | SD | % | SD | % | SD | % | SD | % | SD |
| AER CNT | 95 | 6.66 | 100 | 0.32 | 97 | 1.86 | 68 | 6.54 | 77 | 1.25 |
| AC CNT | 98 | 2.13 | 88 | 12.19 | 90 | 8.56 | 97 | 3.66 | 99 | 1.63 |
| AER HA | 93 | 1.01 | 100 | 0.43 | 98 | 2.24 | 68 | 6.35 | 77 | 1.24 |
| AC HA | 29 | 12.70 | 86 | 3.69 | 83 | 3.08 | 58 | 3.31 | 85 | 3.02 |
| AER EDTA | 92 | 2.47 | 99 | 0.79 | 97 | 1.85 | 66 | 4.33 | 77 | 1.19 |
| AC EDTA | 13 | 0.17 | 85 | 5.56 | 97 | 13.38 | 86 | 4.60 | 92 | 1.03 |
| AER SDS | 93 | 1.59 | 100 | 0.42 | 98 | 2.31 | 68 | 6.42 | 77 | 1.25 |
| AC SDS | 18 | 3.12 | 82 | 8.89 | 91 | 11.64 | 94 | 8.09 | 94 | 4.29 |
| AER MgCl2 | 100 | 0.00 | 100 | 0.69 | 98 | 2.01 | 68 | 6.26 | 77 | 1.22 |
| AC MgCl2 | 20 | 0.00 | 92 | 3.05 | 96 |  | 68 | 6.09 | 96 | 6.32 |
| AER KNO3 | 95 | 1.45 | 92 | 3.02 | 97 | 1.77 | 68 | 5.86 | 77 | 1.24 |
| AC KNO3 | 22 | 14.20 | 90 | 4.11 | 95 | 7.45 | 70 | 3.85 | 93 | 1.70 |
| AER Na3PO4 | 98 | 1.71 | 99 | 1.37 | 97 | 1.78 | 68 | 6.48 | 77 | 1.18 |
| AC Na3PO4 | 27 | 5.01 | 85 | 3.82 | 87 | 2.14 | 62 | 0.10 | 77 | 0.97 |
| AER NaHCO3 | 89 | 3.13 | 99 | 1.90 | 96 | 1.47 | 66 | 3.44 | 77 | 1.00 |
| AC NaHCO3 | 16 | 5.48 | 85 | 3.82 | 90 | 2.41 | 68 | 6.42 | 100 | 0.11 |

**Table S11.** Initial (0 h) and final (24 h) solution pH measured during adsorption experiments of five PFAS in the presence of seven co-occurring ions: HA -EDTA (red), PFAS-SDDS MgCl_2_, KNO_3_, Na_3_PO_4,_ NaHCO₃ along the controls on GAC and AER. Standard deviation of duplicate measurements.

| Experiment | 0 Hour | | 24 Hour | |
| --- | --- | --- | --- | --- |
|  | pH | SD | pH | SD |
| AER CNT | 3.49 | 0.13 | 4.47 | 0.50 |
| AC CNT | 3.93 | 0.32 | 7.62 | 0.28 |
| AER HA | 3.59 | 0.02 | 4.01 | 0.13 |
| AC HA | 3.60 | 0.21 | 6.22 | 0.87 |
| AER EDTA | 3.94 | 0.40 | 4.05 | 0.02 |
| AC EDTA | 3.72 | 0.18 | 6.94 | 0.08 |
| AER SDS | 3.71 | 0.47 | 4.13 | 0.06 |
| AC SDS | 3.94 | 0.09 | 7.08 | 0.11 |
| AER KNO3 | 4.03 | 0.38 | 4.08 | 0.02 |
| AC KNO3 | 4.35 | 0.49 | 7.72 | 0.30 |
| AER Na3PO4 | 9.99 | 0.30 | 7.6 | 0.52 |
| AC Na3PO4 | 9.79 | 0.06 | 9.75 | 0.19 |
| AER NaHCO3 | 9.71 | 0.29 | 7.61 | 0.14 |
| AC NaHCO3 | 9.44 | 0.08 | 9.52 | 0.22 |
| AER MgCl2 | 4.02 | 0.02 | 4.08 | 0.02 |
| AC MgCl2 | 4.40 | 0.08 | 7.37 | 0.07 |

**References**

Fatima, M., Kelso, C., & Hai, F. I. (2026). Beyond the isotherm: Limits in prediction of PFAS breakthrough behaviour in continuous flow column. *Journal of Water Process Engineering*, *81*, 109352. <https://doi.org/https://doi.org/10.1016/j.jwpe.2025.109352>

1. ^a^ Strategic Water Infrastructure Laboratory, School of Engineering, University of Wollongong, Wollongong, NSW, 2522, Australia.

   ^b^ Molecular Horizons, School of Science, University of Wollongong, Wollongong, NSW, 2522, Australia.

   *** Corresponding Author**: Tel.: +61-2-42213054. Email: [faisal@uow.edu.au](mailto:faisal@uow.edu.au) (H. Faisal)
